# Peritumoral Immune-suppressive Mechanisms Impede Intratumoral Lymphocyte Infiltration into Colorectal Cancer Liver versus Lung Metastases

**DOI:** 10.1158/2767-9764.CRC-23-0212

**Published:** 2023-10-12

**Authors:** Jian Ye, Weihua Guo, Chongkai Wang, Colt A. Egelston, Massimo D'Apuzzo, Geereesh Shankar, Marwan G. Fakih, Peter P. Lee

**Affiliations:** 1 Department of Immuno-Oncology, Beckman Research Institute of the City of Hope, Duarte, California.; 2 Department of Medical Oncology and Therapeutics Research, City of Hope National Medical Center, Duarte, California.; 3 Department of Pathology, City of Hope National Medical Center, Duarte, California.; 4 College of Medicine, University of Cincinnati, Cincinnati, Ohio.

## Abstract

Patients with microsatellite stable (MSS) colorectal cancer with liver metastases are resistant to immune checkpoint inhibitor (ICI) therapy, while about one-third of patients with colorectal cancer without liver metastases, particularly those with lung-only metastases, respond to ICI. We analyzed primary colorectal cancer tumors and major metastatic sites (liver, lung, peritoneal) using multiplex immunofluorescence and whole-slide spatial analyses to identify variations in immune contexture and regional localization within the tumor microenvironment. While levels of T and B cells within peritumoral regions were similar, their levels were significantly lower within the tumor core of liver and peritoneal metastases compared with lung metastases. In contrast, antigen-presenting cells (APC) and APC–T cell interactions were more abundant in all regions of lung metastases. We also identified an abundance of lymphoid aggregates throughout lung metastases, but these were present only within peritumoral regions of liver and peritoneal metastases. Larger lymphoid aggregates consistent with features of tertiary lymphoid structures were observed within or adjacent to primary tumors, but not metastatic lesions. Our findings were validated using NanoString GeoMx DSP, which further showed that liver metastases had higher expression of immune-suppressive markers, while lung metastases showed higher proinflammatory activity and T-cell activation markers. Peritoneal metastases demonstrated higher expression of cancer-associated fibroblast–related proteins and upregulated PD-1/PD-L1 signaling molecules. Our results demonstrate that functional status and spatial distribution of immune cells vary significantly across different metastatic sites. These findings suggest that metastatic site–dependent immune contexture may underlie discordant responses to ICI therapy in patients with MSS colorectal cancer.

**Significance:**

Our results demonstrate that functional status and spatial distribution of immune cells vary significantly across different metastatic sites in MSS colorectal cancer. These findings suggest that metastatic site–dependent immune contexture may underlie discordant responses to ICI therapy in patients with MSS colorectal cancer.

## Introduction

Colorectal cancer is the third most common cancer worldwide, and metastasis is the leading cause of colorectal cancer–related death. Colorectal cancer cells can metastasize to the liver, lung, peritoneum, distant lymph nodes, ovaries, bone, and brain. Overall survival differs by sites of metastatic disease, with specific metastatic sites being recognized as negative prognostic factors in patients with metastatic colorectal cancer (1). In recent years, immunotherapy using immune checkpoint inhibitors (ICI) has shown efficacy in patients with colorectal cancer with microsatellite instability-high tumors, but patients with colorectal cancer with microsatellite stable (MSS) tumors respond poorly to ICI treatment (2, 3). We and other groups have shown that patients with MSS colorectal cancer may still benefit from immunotherapy via a combination of ICI and multitargeted tyrosine kinase inhibitors (TKI). However, responses to this combination therapy are restricted to patients without hepatic metastases at time of treatment (4–6). In the U.S. REGONIVO study, 70 patients with MSS colorectal cancer were treated with the PD-1 inhibitor nivolumab and a multikinase inhibitor, regorafenib (6–8). Objective response rate in 23 patients without liver metastatic disease was 22%. In contrast, none of the 47 patients with liver metastatic disease experienced a response. In the trial of regorafenib, ipilimumab, and nivolumab (RIN), of 22 patients with non-liver metastatic disease, up to 36% of patients responded to immuno-oncology (IO) therapy (9). In addition, a recent study exploring the combination of CTLA-4 inhibitor botensilimab and PD-1 inhibitor balstilimab reported an overall response rate of 42% in MSS patients without liver metastatic disease versus 0% in patients with liver metastases (10). These clinical observations led us to hypothesize that organ-specific tumor microenvironment (TME) differences may impact the overall survival of patients with colorectal cancer and clinical outcome of immunotherapies.

Liver is the most common site for metastasis in colorectal cancer. Approximately half of patients with colorectal cancer develop liver metastases, leading to two-thirds of deaths in patients with colorectal cancer (11). The liver is considered to be an immunologically tolerant organ, partly because the hepatic portal system drains blood with food-derived antigens and microbial products from the intestinal tract, thus requiring limited activation of immune responses to foreign antigens (12). While the liver can mount an initial immune response to inflammation or injury, immunosuppressive components within the liver may promote metastases both locally and systemically (13). Mechanistic animal studies demonstrated that liver metastases can suppress systemic antitumor immunity due to activation of regulatory T cells (Treg; ref. 14) or depletion of CD8^+^ T cells by hepatic macrophages (15).

Lung is the second most common metastatic site of colorectal cancer. Lung metastases are known to have high levels of immune effector cell infiltration and antigen presentation regardless of primary tumor of origin (16). As parts of the common mucosal immune system, lung and gut share similarities and may communicate with each other through inflammatory cytokine stimulation, immune cell migration, and microbial interactions (17, 18). Therefore, immune responses against primary colorectal cancer tumors may partially shape the TME in lung metastases. Beyond liver and lung, the third most common site for colorectal cancer metastasis is peritoneum. Peritoneal metastases occur on the peritoneal mesothelium which is supported by a thin layer of connective tissue; they are often fibrotic, which may prevent T-cell infiltration and lead to poor prognosis (19, 20). The tumor immune microenvironment in colorectal cancer metastases appears to be strongly influenced by the organ site. However, organ-specific TME features of colorectal cancer metastases have not been well characterized via parallel comparison.

Emerging evidence suggest a strong association between tumor immune infiltration and clinical outcomes. Immunoscore, which assesses the density of CD3^+^ and cytotoxic CD8^+^ T cells within tumor and invasive margin, outcompetes all other clinical parameters in predicting patient outcomes in both primary and metastatic settings (21, 22). In addition, it is not only density of tumor-infiltrating lymphocytes but also their spatial organization that determine antitumor immunity. For example, functional immune cell clusters have a strong prognostic impact on overall survival (23). Lymphoid aggregates (LA) of sufficient size and containing a germinal center are referred to as tertiary lymphoid structures (TLS). TLSs in primary colorectal cancer tumors are associated with favorable clinical outcomes and responses to immunotherapy in other cancers (24, 25). Dense antigen-presenting cell niches, which can recruit T-cell factor-1^+^ (TCF1^+^) stem-like CD8 T cells, were also associated with favorable outcomes, possibly by facilitating T-cell infiltration into the TME (26). Therefore, comprehensive characterization of immune composition by spatial analysis to dissect potential cell–cell interactions may provide insights into biomarkers of response to immunotherapy as well as potential biomarkers of drug resistance.

In this work, we performed multiplex immunofluorescence (mIF) staining on 21 primary and 42 metastatic colorectal cancer tumors and identified 14 immune cell subsets within five histologically defined regions of each site. Pattern of immune cell infiltration, distribution of LAs, and spatial association of T cells with APCs were characterized within each organ-specific TME. Through GeoMx high-plex proteomic analysis, we also show that liver metastases have higher expression of immune-suppressive markers, while lung metastases have higher proinflammatory activity and T-cell activation and peritoneal metastases have higher expression of cancer-associated fibroblast–related proteins and upregulated PD-1/PD-L1 signaling molecules. Our study sheds light on how disparate TME immune characteristics in metastatic sites may drive different clinical outcomes in patients with colorectal cancer.

## Materials and Methods

### Patients

We identified cases of metastatic colorectal cancer tumors, regardless of whether primary tumor resection was performed, from the tumor registry at the City of Hope Comprehensive Cancer Center for the period between 1989 and 2015. Our research involved 42 patients with stage IV colon cancer, all of whom received preoperative chemotherapies, including fluorouracil, capecitabine, and oxaliplatin. This cohort included 2 patients with only primary tumor specimens, 13 patients with synchronously resected primary and liver metastatic tumor specimens, 5 patients with only liver metastatic tumor specimens, 6 patients with paired primary and lung metastatic tumor specimens (one synchronous), 6 patients with only lung metastatic tumor specimens, 8 patients with only peritoneal metastatic tumor specimens, and 2 patients with paired liver and lung metastatic tumor specimens, were selected for the analysis (Supplementary Table S1). In total, 63 primary and metastatic tumors from this cohort were profiled by the multiplex immunohistoflurescence assay.

### Multiplex Immunofluorescence Assay

Formalin-fixed, paraffin-embedded (FFPE) specimens from the City of Hope cohort were cut into 3 µm sections, fixed to microscope slides, and stained with four mIF biomarker panels. The first panel included PD-1 (Cell Marque, NAT105, RRID:AB_1160822; 1:600; Opal 690; pH 9), CD4 (Cell Marque, EP204, catalog no. 104R; 1:3,000; Opal 520; pH 9), CD8 (Leica, 4B11, RRID:AB_10555292; 1:800; Opal 540; pH 9), CD3 (Dako, Polyclonal, catalog no. GA503; 1:1,000; Opal 620; pH 6), FOXP3 (Biocare, 236A/E7, catalog no. API3164AA; Opal 570; pH 6), and Cytokeratin 20 (CK20; Dako, KS20.8, RRID:AB_2133718; 1:800; Opal 650; pH 9). The second panel included PD-L1 (Spring, SP142, RRID:AB_2861330; 1:600; Opal 570; pH 9), CD68 (Biocare, KP1, RRID:AB_2885063; 1:600; Opal 520; pH 9), CD163 (Leica, 10D6, RRID:AB_2920861; 1:800; Opal 690; pH 9), CD66b (Novus, G10F5, RRID:AB_1084038; 1:1300; Opal 540; pH 9), and CK20 (Dako, KS20.8, RRID:AB_2133718; 1:800; Opal 650; pH 9). The third panel included PD-1 (Cell Marque, NAT105, RRID:AB_1160822; 1:600; Opal 690; pH 9), PD-L1 (Spring, SP142, RRID:AB_2861330; 1:600; Opal 570; pH 9), TCRδ (Santa Cruz Biotechnology, H-41, RRID:AB_1130061; 1:800; Opal 540; pH 9), CD20 (Dako, L26, RRID:AB_2282030; 1:450; Opal 520; pH 6), and CK20 (Dako, KS20.8, RRID:AB_2133718; 1:800; Opal 650; pH 9). The fourth panel included HLA-DR (Abcam, TAL1B5, RRID:AB_445401; 1:90,000; Opal 620; pH 6), CD4 (Cell Marque, EP204, catalog no. 104R; 1:3,000; Opal 520; pH 9), BCL6 (Biocare, LN22, RRID:AB_10890175; 1:1,000; Opal 570; pH 9), TCF1 (Cell Signaling Technology, C63D9, RRID:AB_2199302; 1:1,600; Opal 690; pH 9), CD8 (Leica, 4B11, RRID:AB_10555292; 1:800; Opal 540; pH 9), and CD20 (Dako, L26, RRID:AB_2282030; 1:450; Opal 650; pH 6). Biomarker staining and image acquiring were done as reported previously (27). Briefly, the slides were deparaffinized with xylene and rehydrated with decreasing concentrations of ethanol in water. Heat-induced epitope/antigen retrieval was performed in AR9 (pH 9) or AR6 buffer (pH 6; PerkinElmer) using a microwave oven. Blocking was performed for 10 minutes using Antibody Diluent Background Reducing (S3022, Agilent) to minimize nonspecific background staining. Slides were then serially stained with primary antibodies for 1 hour on a shaker at room temperature. Antibodies were then detected by horseradish peroxidase–conjugated secondary antibodies (MACH2 HRP-Polymer, Biocare) and an Opal Fluorescence IHC kit (PerkinElmer) at a 1:100 dilution following a 10-minute incubation. To perform multicolor immunofluorescent staining, the slide was stained in the sequence listed for each panel. Microwave incubation was used to remove previous antibodies, while simultaneously exposing the next epitope of interest. After staining for the final marker, the cell nuclei were stained with DAPI (PerkinElmer). The slides were then mounted with ProLong Gold Antifade Reagent (P36930, Thermo Fisher Scientific).

### Image Scanning and Analysis

The workflow is shown in a flowchart (Supplementary Fig. S1A). Briefly, stained slides were scanned over the whole slide using the Vectra 3.0 system (PerkinElmer), which initially captured the fluorescence spectra in five channels (DAPI, FITC, Cy3, Texas Red, and Cy5) from images obtained with a 10× Olympus lens objective. Using a Phenochart whole-slide reviewer (PerkinElmer), whole tissue was selected and captured with a 20× Olympus lens objective using the Vectra system in the same channels. Images of tissues stained for a single marker and unstained tissues were used to extract the spectrum of each fluorophore and tissue autofluorescence in the 20× images, and to create a spectral library to perform multispectral unmixing using inForm Cell Analysis software (PerkinElmer). The high-power field images were stitched into whole scan images for each tumor specimen with QuPath (28). The compartments of each whole scanned image were manually delineated into five regions, including tumor core (inside 0.5 mm from tumor boundary), inner invasive margin (the boundary to 0.5 mm inside of the tumor), outer invasive margin (the boundary to 0.5 mm outside of the tumor), juxtatumoral region (0.5 to 2 mm outside of tumor), and distal region (5 mm outside of tumor). Training images were generated by selecting and combining > 40 representative regions of interest (ROI) per metastatic site from each mIF panel. The tissue classifiers to distinguish tissue types (cancer island, stroma, necrosis) and cell classifiers to identify markers were then trained and applied to the whole scanned slides with manual region segmentation. The image raw data were exported from QuPath and analyzed with R and Python

### GeoMx Digital Spatial Profiling

For immune profiling of organ-specific metastases in patient samples, the GeoMx Digital Spatial Profiler (NanoString), a custom-built high-speed automated system and integrated instrument software, was used. We analyzed five samples from each organ that exhibited typical immune cell distribution, as characterized by mIF. A multiplexed cocktail of primary antibodies with UV photocleavable indexing oligonucleotides (GeoMx Immune Profile Core; 22 targets, including three isotype controls and three additional modules: IO Drug Target, Immune Activation Status, and Immune Cell Typing) and two fluorescent markers was applied to a slide-mounted FFPE tissue section. For the fluorescent markers, we used CD45-AF647 (Novus, C3e/1308; 1:100) and pan-CK-Dy550 (Novus, HMB45; 1:100). Images at 20× magnification were assembled to yield a high-resolution image of the tissue area of interest. The specific ROIs for molecular profiling were then selected on the basis of location and sequentially processed by the microscope automation. ROIs were selectively illuminated with UV light to release the indexing oligos by coupling UV LED light with a double digital mirror device module. Following each UV illumination cycle, the eluent was collected from the local region via microcapillary aspiration and transferred to an individual well of a microtiter plate. Once all ROIs were processed, pools of released indexing oligos were hybridized to NanoString optical barcodes for digital counting and subsequently analyzed with a nCounter Analysis System. nCounter hybridization assay for photocleaved oligo counting Hybridization of cleaved indexing oligonucleotides to fluorescent barcodes was performed using the nCounter Protein PlexSet reagents based on the manufacturer's directions. Hybridizations were performed at 65 °C overnight in a thermocycler. After hybridization, samples were processed using the nCounter Prep Station and Digital Analyzer as per manufacturer instructions. Data were normalized to technical controls and areas. Data were calculated against isotype controls to generate signal-to-noise ratios. Protein targets with a signal-to-noise ratio less than 2 were removed from downstream analysis.

### Statistical Analysis

Wilcoxon rank-sum tests were used to determine the difference between each pair of metastatic sites. The Kaplan–Meier method, in conjunction with the log-rank test, was used to analyze the overall survival of colorectal cancer patient groups, each with different organ involvement. To adjust the *P* values for multiple testing, we employed the Benjamini and Hochberg method. The *χ*^2^ test was utilized to examine the percentage of tumor samples with intratumoral LA. Datapoints represent mean values, and error bars represent SE. Statistical significance is indicated by asterisks: *, *P* < 0.05; **, *P* < 0.01; ***, *P* < 0.001; ****, *P* < 0.0001. All analyses and data visualization were performed in Python 3.8.8 and R 4.2.2.

### Study Approval

The study using the City of Hope cohort was conducted under an Institutional Review Board–approved protocol (IRB18198).

### Data Availability

All data relevant to the study are included in the article or uploaded as Supplementary Data. Raw data and scripts are available from the corresponding author upon reasonable request.

## Results

### Histologic Classification and Variations Across Colorectal Tumors and Organ-specific Metastases

**FIGURE 1:**
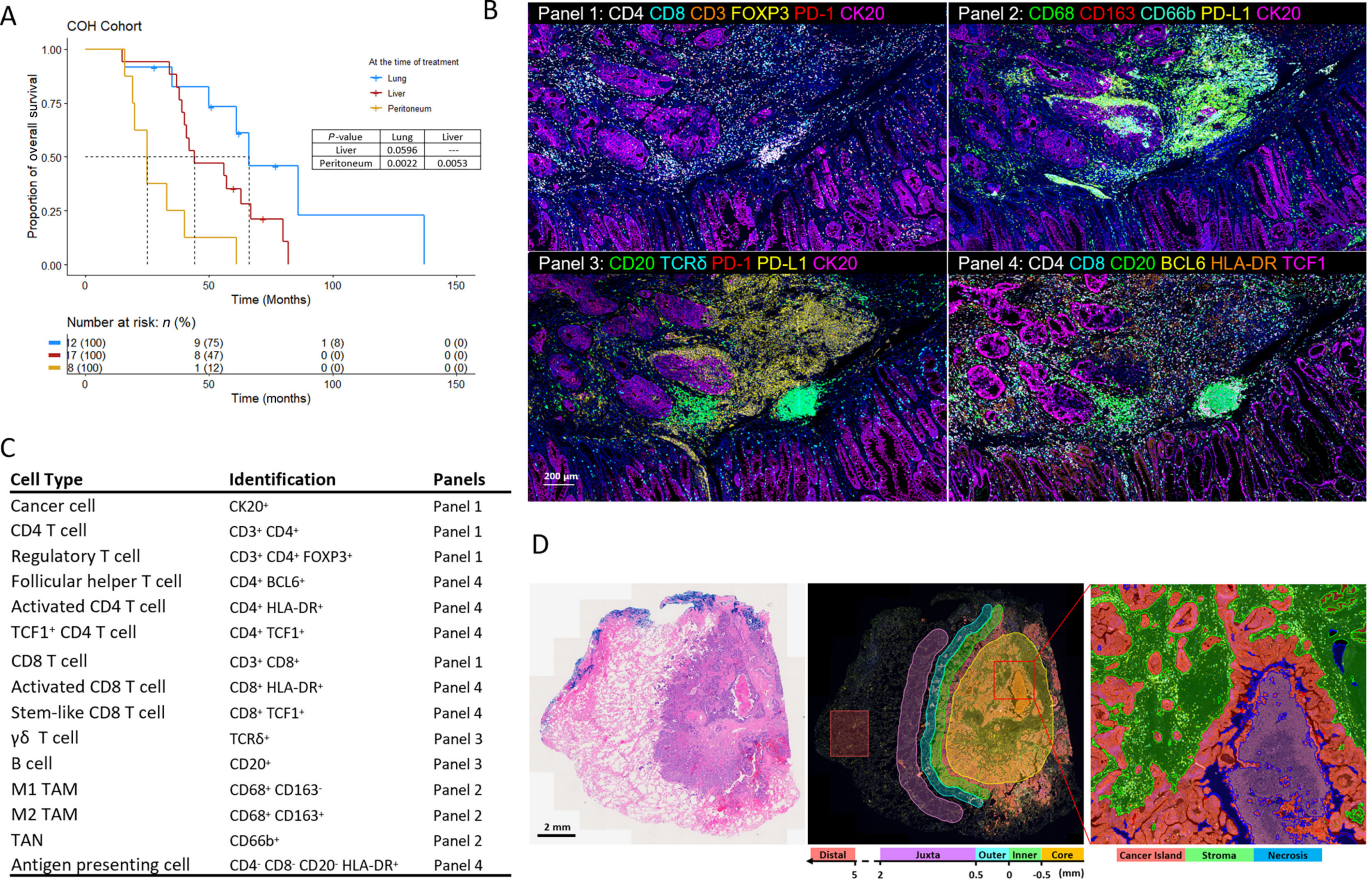
Metastatic colorectal cancer tumor specimens and cell population evaluated by mIF. **A,** Kaplan–Meier curve of overall survival in patients with colorectal cancer with metastases in liver (*n* = 17), lung (*n* = 12), and peritoneum (*n* = 8). Numbers below each *x* axis indicate the number of patients at risk and those in parentheses are the number of events. Statistical significance was determined by log-rank test (**B**). Representative images showing four panels of mIF staining from consecutive sections of a colorectal cancer primary tumor. **C,** Cell lineages identified by hierarchical gating of lineage and functional biomarkers during image analysis of mIF staining. **D,** Representative images of a colorectal cancer lung metastasis showing manual segmentation of five compartments (tumor core, inner invasive margin, outer invasive margin, juxta tumor, and distal tissue) and tissue segmentation by machine learning algorithm (cancer island, stroma, and necrosis).

We conducted a comprehensive characterization of 21 archival resected primary tumors, 20 liver metastases, 12 lung metastases, and 8 peritoneal metastases from 42 patients with MSS colorectal cancer. A total of 13 patients with liver metastases and 6 patients with lung metastases had matched primary and metastatic tumor specimens (Supplementary Table S1). Overall survival of patients in this study was consistent with previous reports (19), showing that patients with lung metastases had the best survival, followed by liver metastases, and then peritoneal metastases (Fig. 1A).

Four consecutive FFPE tissue sections per tumor were subjected to mIF staining with antibody panels designed to identify T cells (Panel 1), myeloid cells (Panel 2), B cells, and γδ T cells (Panel 3), and interaction between T-cell subsets and APCs (Panel 4) (Fig. 1B). Scanned digital images were quantitatively analyzed and 14 immune subsets were quantified, including total CD4 and CD8 T cell and their subsets (FOXP3^+^ Tregs, BCL6^+^ T follicular helper T cells (Tfh) HLA-DR^+^ activated T cells, and TCF1^+^ T cells), B cells, γδ T cells, M1/M2 tumor-associated macrophages (TAM), tumor-associated neutrophils (TAN), and APCs (Lin-HLA-DR^+^; Fig. 1C; Supplementary Fig. S1A). T cells and B cells were further evaluated for expression of programmed death-1 receptor (PD-1). TAMs and neutrophils were evaluated for expression of programmed death-ligand 1 (PD-L1).

In most tumor samples, we observed uneven distribution of immune cells in different histologic regions. Lymphocytes were enriched at the outer invasive margins, especially in liver metastases, suggesting a migration block or lack of lymphocyte recruitment into the tumor parenchyma (Supplementary Fig. S1B). To better compare the cell densities among primary tumors and different organ-specific metastases, we manually segregated whole-slide images into five regions (29) tumor core, inner and outer invasive margin, juxta tumor, and distal region (Fig. 1D). To generate unbiased quantitative data, we utilized a machine learning algorithm to further differentiate the tumor core and inner invasive margin into three subregions: cancer island, stroma, and necrosis, based on cancer cell occupancy and nuclear morphology (Fig. 1D; Supplementary Fig. S1B). Proportions of each subregion within primary and metastatic tumors were largely comparable, with an increased percentage of necrotic tissue in peritoneal metastases (Supplementary Fig. S1C). We observed higher immune cell density within stroma than cancer islands, and heterogeneous immune cell infiltration in necrotic regions of individual samples.

### Primary Tumors and Lung Metastases Display Enhanced Lymphocyte Infiltration and APC Presence

**FIGURE 2:**
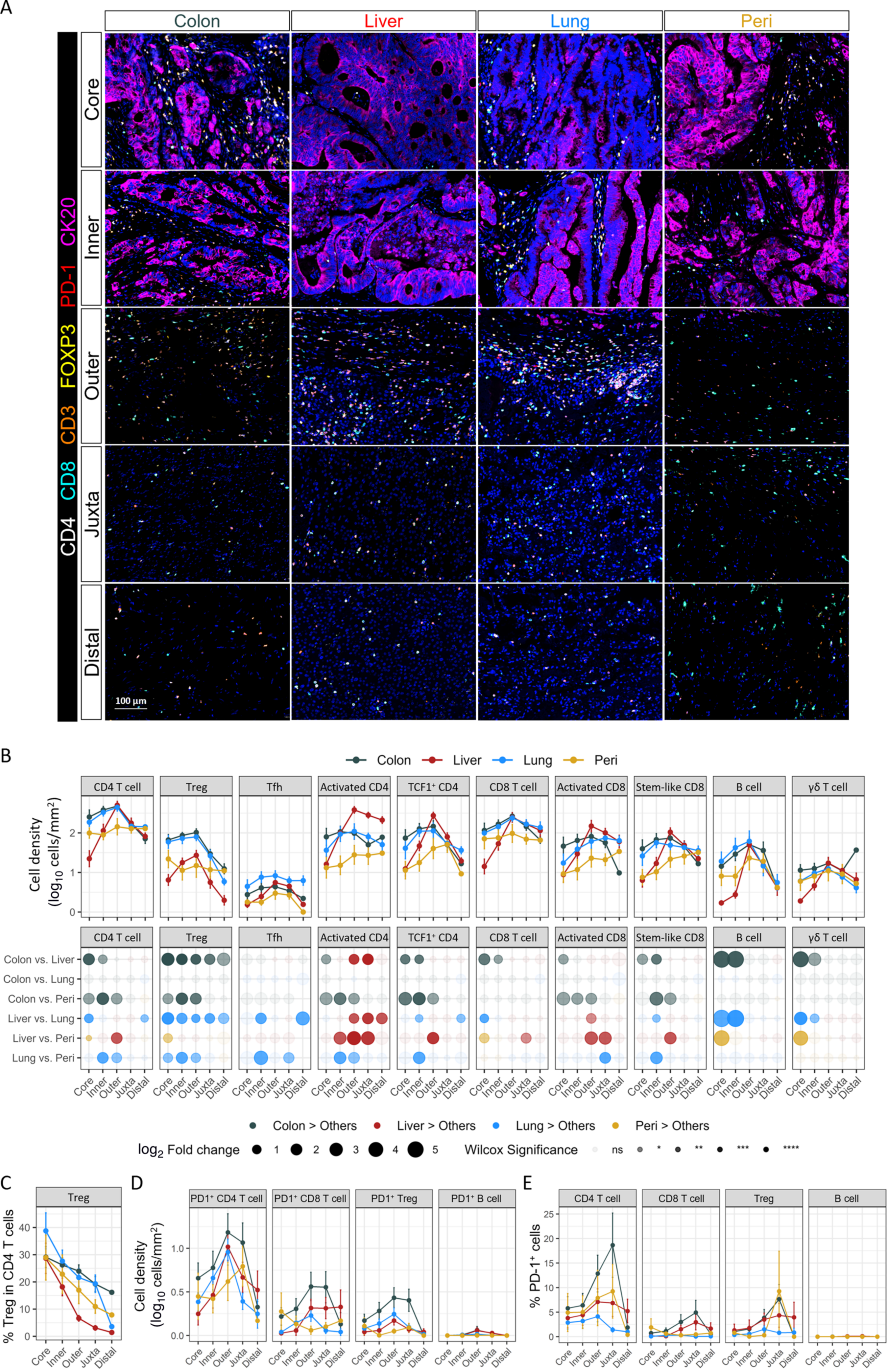
Different distribution of lymphocytes in diverse tumor regions. **A,** Representative images of primary and metastatic colorectal cancer tumors stained with mIF panel 1, five images in each column were selected from the same tumor. **B,** Cell density (log_10_ cells/mm^2^) of lymphocytes in the TME in different histopathologic regions (Core, Inner, Outer, Juxta and Distal). Paired comparison of lymphocyte density between each two organs in the different histologic regions shown in bottom row. Statistical significance was determined by Wilcoxon signed-rank test. Percentage of Foxp3^+^ Tregs within CD4 T cells (**C**), cell density (log_10_ cells/mm^2^) of PD-1–positive lymphocytes in TME (**D**), and percentage of PD-1–positive cells within indicated lymphocytes (**E**) in different histopathologic regions. Paired comparison between primary and metastatic tumor sites are in Supplementary Fig. S3A–S3C.

The tumor core and inner invasive margin of primary tumors and lung metastases have higher lymphocyte infiltration (CD4 and CD8 T cells, B cells, and γδ T cells) than in liver and peritoneal metastases. In contrast, densities of lymphocytes were largely comparable within the outer invasive margin, juxta tumor, and distal region across all metastatic sites, except for less T cells in the outer invasive margin of peritoneal metastases (Fig. 2A and B; Supplementary Fig. S2A). We further quantified CD4 and CD8 T-cell subsets identified by expression of unique transcriptional factors or surface markers (Fig. 1C; Supplementary Fig. S1D). Tregs were more abundant in all regions of primary tumor and lung metastases. The percentage of Treg within total CD4 T cells gradually reduced from tumor core to distal region among all sites, demonstrating an enrichment of Treg within tumor cores (Fig. 2C; Supplementary Fig. S3A). T follicular helper T cells (Tfh) are critical for efficient antibody responses by B cells. Their presence is often associated with better outcomes in solid tumors including colorectal cancer (30, 31). We found that Tfh were more abundant within lung metastatic tumors than other sites (Fig. 2B; Supplementary Fig. S2B). Consistent with the overall density of CD4 and CD8 T cells, densities of HLA-DR^+^ activated T-cell subsets, which are major producers of cytotoxic molecules and proinflammatory cytokines (32), were also lower within the tumor core of liver metastases. In contrast, activated CD4 T cells were significantly enriched in the outer invasive margin of liver metastases. This spatial gradient suggests deficient tumor infiltration of T effectors within liver metastases (Fig. 2B). Transcription factor TCF1 plays a critical role in the regulation of T-cell development, stemness, and memory formation. TCF1^+^ CD4 T cells have been identified as a population with memory-like features that can self-renew and give rise to both activated T cells and Tfh (33, 34). Concurrently, TCF1^+^ CD8 stem-like T cells, which are generated in response to either viral or tumor antigens, play a crucial role in preserving CD8 T-cell infiltration in the TME (26, 35, 36). We observed that TCF1^+^ CD4 T cells and CD8 stem-like T cells were similarly distributed as total CD4 and CD8 T cells, with more intratumoral infiltration into primary tumors and lung metastases (Fig. 2B). In addition, primary tumors and metastases had comparable cell densities and percentages of PD-1^+^ T cells and B cells, with a slight increase within primary tumors. More PD-1^+^ T cells are found in the peritumoral regions of primary tumors (Supplementary Fig. S4). Even though the density of Treg in the tumor core of liver metastases is lower compared with that in primary tumor and lung metastases (Fig. 2D; Supplementary Fig. S3B), a higher proportion of PD-1^+^ cells can be found within Treg in liver metastases (Fig. 2E; Supplementary Fig. S3C).

**FIGURE 3:**
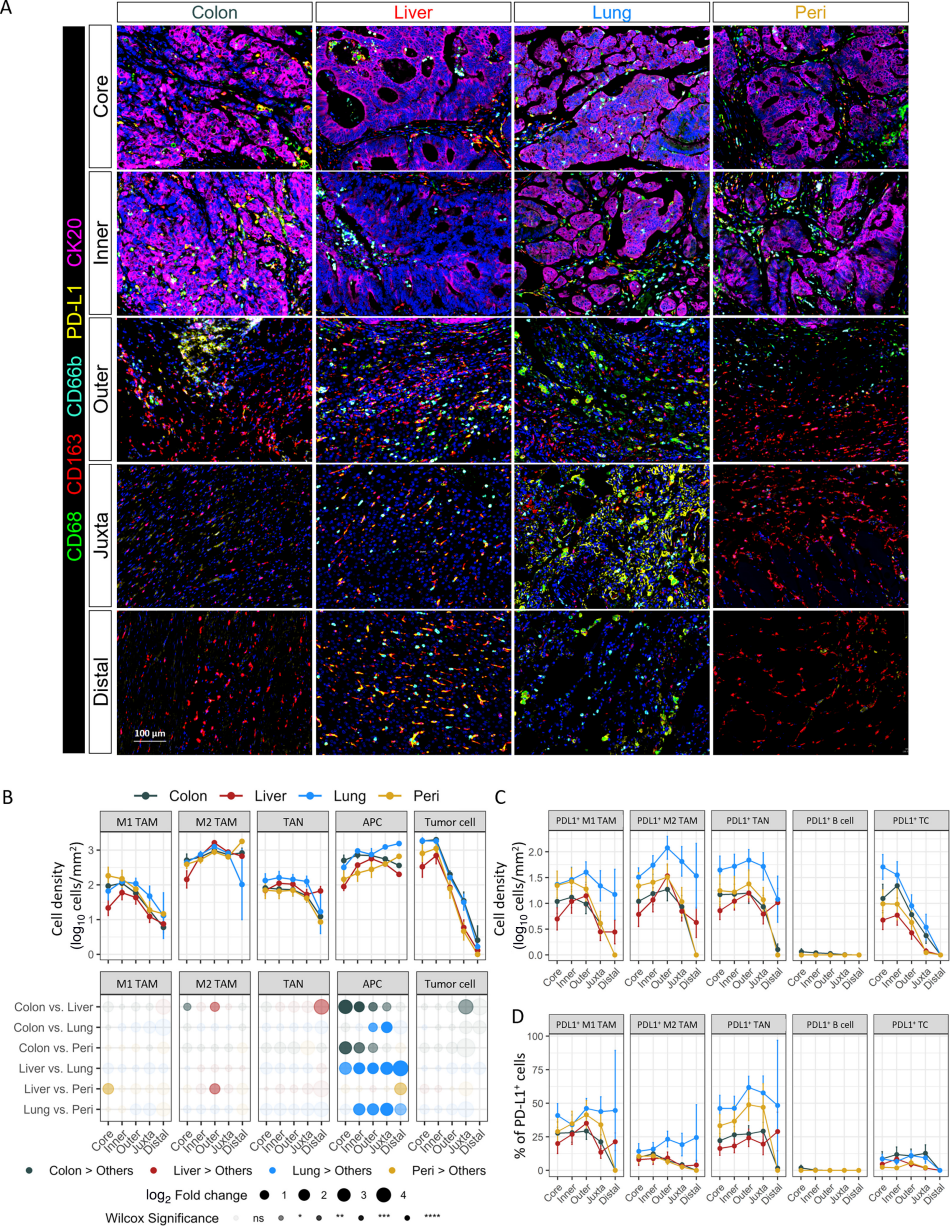
Distribution of myeloid cells in diverse tumor regions. **A,** Representative images of primary and metastatic colorectal cancer tumors stained with mIF panel 2, five images in each column were selected from the same tumor. **B,** Cell density (log_10_ cells/mm^2^) of myeloid cells and tumor cells in the TME in different histopathologic regions (Core, Inner, Outer, Juxta and Distal). Paired comparison of cell density between each two organs in the different histologic regions shown in bottom row. Statistical significance was determined by Wilcoxon signed-rank test. Cell density (log_10_ cells/mm^2^) of PD-L1–positive cells in TME (**C**) and percentage of PD-L1–positive cells within indicated cell types (**D**) in different histopathologic regions. Paired comparison between primary and metastatic tumor sites are in Supplementary Fig. S3D and S3E.

For myeloid cells, we observed that densities of M1/M2 TAMs and TANs were largely comparable within most regions of primary and metastatic tumors, except for more M2 TAM in the outer invasive margin of liver metastases (Fig. 3A and B). In contrast, there were significantly more APCs within all regions of primary tumors and lung metastases compared with liver and peritoneal metastases. We determined PD-L1 expression on myeloid cells, B cells, and tumor cells across all tumor sites. While not significant, there were generally more PD-L1^+^ TAMs and TANs in lung metastases than other sites (Fig. 3C; Supplementary Fig. S3D). Overall, the percentage of PD-L1^+^ cells within myeloid populations was comparable across all tumor sites (Fig. 3D; Supplementary Fig. S3E).

We also separately analyzed immune cell infiltration in cancer islands, stroma, and necrotic tissue within the tumor core and inner invasive margin. Lymphocytes and APCs within cancer islands and stroma were more abundant in primary tumors and lung metastases, with higher cell density in stroma compared with cancer islands. However, necrotic tissue did not differ in immune composition between tumors (Supplementary Figs. S5 and S6). Overall, these results suggest that lymphocyte recruitment into liver metastases is blunted, while antigen presentation is more robust within primary tumors and lung metastases.

### Spatially Distanced T Cells and APCs in Liver Metastases

**FIGURE 4:**
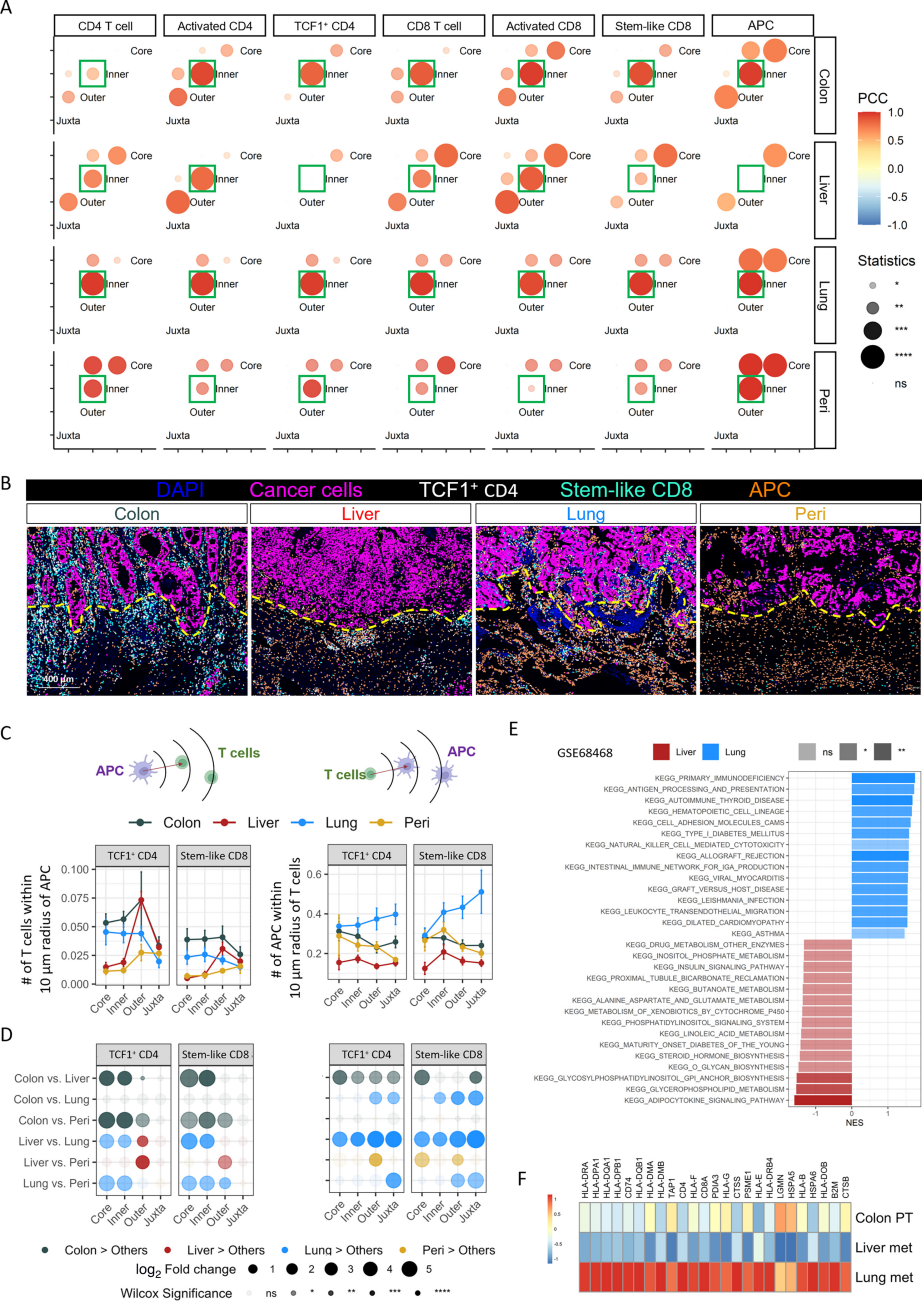
Higher antigen presentation potential in lung metastases. **A,** Correlations of the immune cell density between histologic regions. PCC were calculated from the density of each immune cell type in different histologic regions. **B,** Representative images of primary and metastatic colorectal cancer tumors stained with mIF panel 4. The cancer cells, TCF1^+^ CD4 T cells, stem-like CD8 T cells and APCs were color coded and identified as Fig. 1C. The yellow dash lines indicate the boundaries between inner and outer invasive margin. **C,** Mean number of TCF1^+^ CD4 T cells and stem-like CD8 T cells around 10 µm radius of APCs and mean number of APCs around 10 µm radius of TCF1^+^ CD4 T cells and stem-like CD8 T cells. **D,** Paired comparison of cell density between each two organs in the different histologic regions. Statistical significance was determined by Wilcoxon signed-rank test. **E,** GSEA of liver and lung metastases samples from GES48468. Kyoto Encyclopedia of Genes and Genomes (KEGG) databases were used to determine significantly modified pathways. Bars in red and blue represent, respectively, a positive and negative enrichment in the associated pathway. The *x* axis shows the normalized enrichment score (NES) of the analysis, and the *y* axis the enriched pathways. **F,** Heat map of top genes differentially expressed in GES48468 primary and metastatic colorectal cancer tumor samples from KEGG_ANITGENE_PROCESSING_AND_PRESENTATION pathway.

Gradients in immune cell density across the tumor border may reflect the ability of immune cells to infiltrate from outside to inside tumors. Decreased lymphocyte density within the tumor border of liver metastases, as compared with primary tumor and other metastatic sites, suggests an impaired ability of lymphocytes to infiltrate into the tumor core (Fig. 2B). To assess how immune cell localization is directly impacted by tumor boundaries, we calculated the Pearson correlation coefficient (PCC) of cell densities between two distinct regions within samples from each organ. A higher PCC reflects consistent and unvarying influences impacting cell localization, while a lower PCC suggests more complex factors are involved. In primary tumors, density of most immune cell types correlated well between inner and outer invasive margin (Fig. 4A and B; Supplementary Fig. S7A, highlighted with green boxes). Lung metastases showed strong correlation between densities of T cells, B cells, and dendritic cells (DC) across tumor borders, but a weaker correlation for TAMs and TANs. Liver metastases, on the other hand, showed a lower correlation in the densities of CD4 and CD8 T cells across tumor borders. Liver metastases TCF1^+^ CD4 T cells and stem-like CD8 T-cell subsets showed no correlation between outer and inner invasive margin. In addition, there was no correlation in densities of APCs between outer and inner invasive margin in liver metastases. Peritoneal metastases also showed low correlation in T cells, especially in CD8 T cells, but with a high PCC in APCs. These results suggested that the translocation into the tumor core of APCs and stem-like CD8 T cells, which are necessary for long-term maintenance of T-cell responses and efficacy of immunotherapy (26, 35, 36) is modulated by a multitude of intricate factors in liver metastases.

To further investigate the spatial relationship between T cells with APCs, we quantified the potential availability of APCs and T cells to each other, by determining the number of TCF1^+^ CD4 T cells and stem-like CD8 cells within the direct-contact radius (10 µm) of each APC, as well as the number of APCs close to T cells (Fig. 4C and D). Within the tumor core and inner margin, more TCF1^+^ CD4 T cells and stem-like CD8 cells spatially localized with APC in primary tumors and lung metastases compared with liver and peritoneal metastases, but these were largely comparable in the outer invasive margin and juxta tumor regions. In contrast, fewer APCs were in close proximity to T cells in all regions of liver metastases compared with lung metastases, likely due to poor T-cell infiltration. Similar results were observed when we calculated the mean distance and percentage of cells with direct contacts (Supplementary Fig. S7B and S7C). We also found that while interactions between stem-like T cells and APCs were consistent across all tumor areas in primary tumors and lung metastases, these interactions were significantly lower in the inner invasive margin and tumor core in liver metastases (Supplementary Fig. S7D). In addition, we observed more activated T cells around APCs in liver metastases’ outer invasive margin and juxtatumoral region compared with other metastatic organs, consistent with their enrichment in these areas (Supplementary Fig. S7E; Fig. 2B). We validated the reduced antigen presentation potential in liver metastases by gene sets enrichment analysis (GSEA) in the GSE68468 dataset (37). Consistently, immunologic pathways, including antigen processing and presentation, leukocyte transendothelial migration, and T-cell receptor signaling pathway were enriched in primary colorectal cancer tumors and lung metastases as compared with liver metastases (Fig. 4E; Supplementary Fig. S8A and S8B). We further examined the top genes in antigen presentation pathways. Strikingly, HLA genes were significantly decreased in liver metastases, consistent with a lower abundance of APCs (Fig. 4F).

### Differential TLS Maturation and Distribution Across Primary and Metastatic Colorectal Cancer Tumors

**FIGURE 5:**
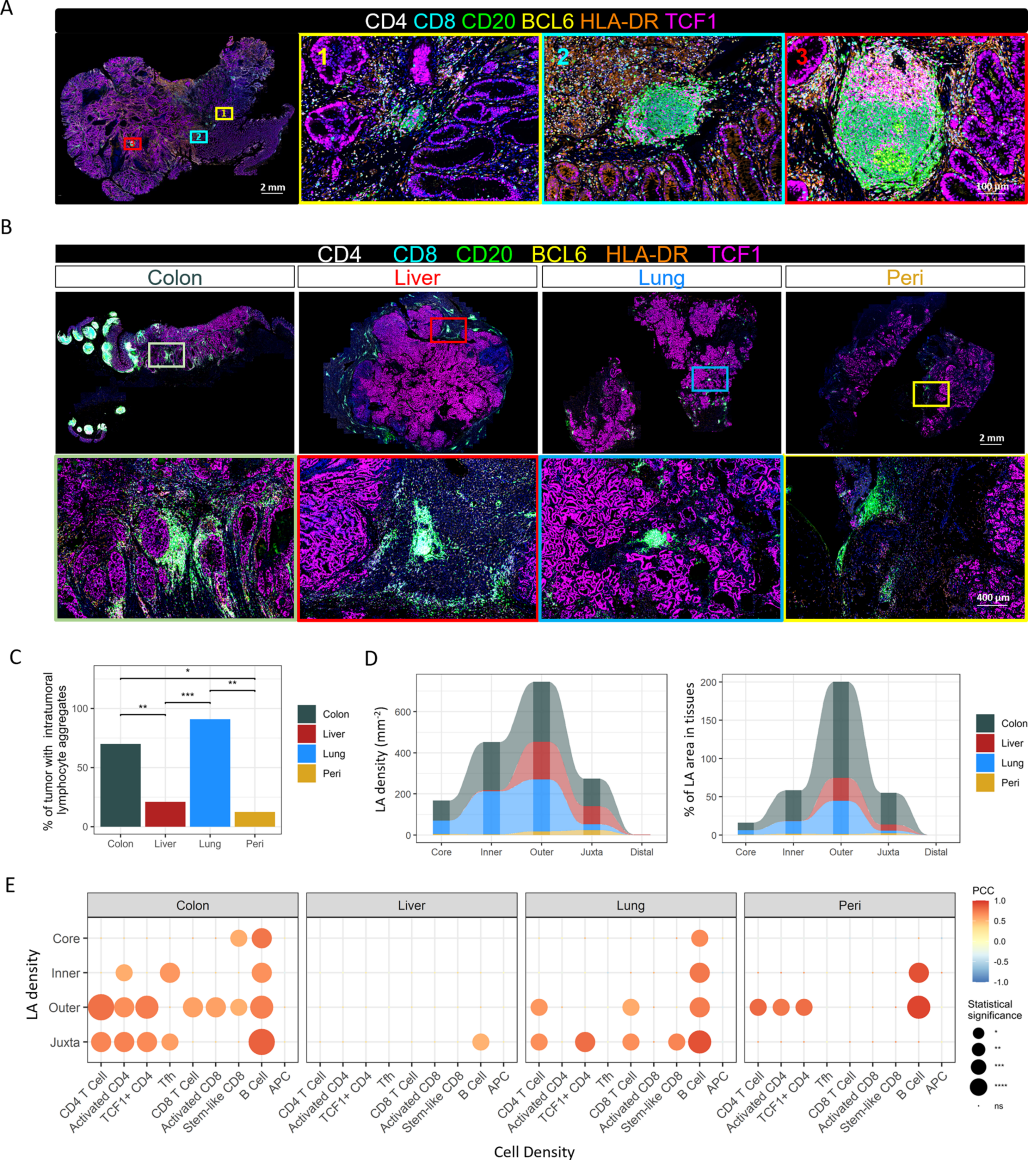
Distribution of LA and TLS across colorectal cancer sites. **A,** Representative images of primary colorectal cancer tumor. Numbered insets show higher magnification. The second inset magnifies the same area seen in Fig. 1B. **B,** Representative images of primary and metastatic colorectal cancer tumors stained with mIF Panel 4. The boxes highlight the LA in the primary and metastatic tumors. **C,** Percentage of tumor specimens with intratumoral LA in primary and metastatic tumors. Statistical significance was determined by *χ*^2^ test. **D,** The number and percentage of area of LA in the metastatic tumor calculated in five individual compartments. **E,** Correlations of LA and various immune cells. PCC were calculated from the density LA and sporadic immune cells in different histologic regions from primary and metastatic colorectal cancer tumors from mIF Panel 4.

TLSs have been reported as favorable prognostic markers for colorectal, lung, breast, and pancreatic cancers (24, 25). Presence of TLS has also been associated with robust immune activation and improved responses to cancer immunotherapy and survival in melanoma and soft-tissue sarcoma (38–41). Mature TLSs are large LAs composed of follicular DCs, B-cell follicles, and adjacent T-cell zone (24, 25, 41). In colorectal cancer, we observed various developmental stages of TLS within/around most primary tumors, from small LAs to mature TLSs which are characterized by the presence of a germinal center with BCL6^+^ and PD-1^+^ Tfh and CD11C^+^ DCs (Fig. 5A; Supplementary Fig. S9A). CD4 and CD8 T cells within the T-cell zone of TLSs were predominantly TCF1^+^ subsets (Supplementary Fig. S9B), consistent with the critical roles of TCF1 in the differentiation of Tfh, memory T cells, and stem-like CD8 T cells (refs. 42, 43; Fig. 5A).

Our study revealed the presence of mature TLS in eight of the 21 studied primary colorectal cancer tumors, a feature not observed in metastatic tumors. We did, however, find small LAs in metastatic tumors, which lacked BCL6^+^ germinal center and could not be defined as mature TLS. Intratumoral LAs were more prevalent within lung metastases in comparison with liver or peritoneal metastases, while the latter had most LAs within the peritumoral regions (Fig. 5B and C). To quantify the distribution and size of LAs, we used a machine learning algorithm to identify LAs within tumors (Supplementary Fig. S10A). Few LAs were found within the tumor core or inner invasive margin of liver or peritoneal metastases, as compared with primary tumors and lung metastases. In contrast, LAs within the peritumoral region, especially at the LA-enriched outer invasive margin, were comparable among primary tumors, liver metastases, and lung metastases. In peritoneal metastases, LAs were rare both inside and outside regions of tumors (Fig. 5D; Supplementary Fig. S10B and S10C). Formation of TLS has been associated with the presence of antigen-specific T-cell responses, such as immune cell recruitment, antigen engagement, and T-cell proliferation (44). Therefore, we determined whether density of tumor-infiltrating immune subsets is associated with abundance of LAs within tumors (Fig. 5E). More regions within primary tumors and lung metastases than liver and peritoneal metastases showed positive association between the number of LAs and densities of nonaggregated T-cell subsets and B cells. In contrast, LA densities were not correlated with APC cell densities in any areas of any tissue type, suggesting that LAs do not drive APC accumulation in tumor tissues. However, LA densities were correlated with B-cell densities in all areas of primary tumors and lung metastases. While CD4^+^ activated T cells and Tfh correlated with LAs within the inner invasive margin of primary tumors, no distinct T-cell subset correlated with LAs in the outer or inner invasive margins of lung metastases. Overall, these results suggest that metastatic tumors may generate weaker immune activity against tumor antigens compared with primary tumors. Among metastatic sites, lung metastases appeared to recruit and activate immune cells more efficiently than other metastatic sites.

### Correlation of T-cell Density in the Tumor Core of Paired Primary and Liver Metastatic Tumors

**FIGURE 6:**
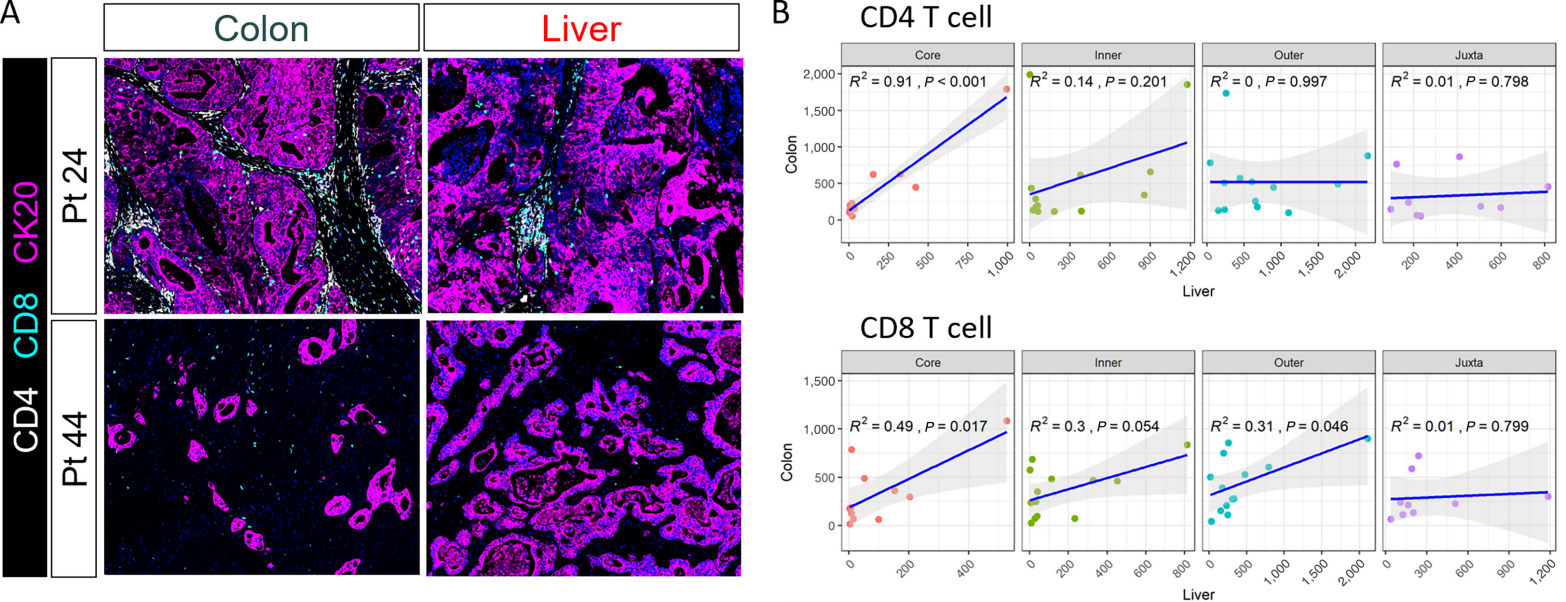
Analysis of immune cells between paired primary and liver metastases. **A,** Representative images of the tumor core from two individual patient's paired primary colorectal cancer and metastatic liver tumors. **B,** Correlations of CD4 and CD8 T cells in the paired primary and liver metastases. *R*^2^ were calculated with the density of immune cells in different histologic regions.

Our patient cohort consisted of 13 patients with synchronously resected primary and liver metastatic tumors, which allowed us to investigate the correlation of immune cell densities within each region of these paired tumor specimens. While liver metastases contain lower levels of immune cells as already noted, both CD4 and CD8 T cells showed high correlations between primary tumors and metastatic samples within the tumor core, but not in other regions (Fig. 6). In contrast, other immune cell populations showed no correlation between primary and metastatic tumors (Supplementary Fig. S11A). This is consistent with recent studies also showing correlation of T-cell density in paired primary tumors and liver metastases (45, 46). This cell type–specific and tumor region–specific correlation may reflect infiltration of tumor antigen–specific T cells into the tumor core (47, 48), while the immune cell compositions within adjacent noncancerous regions may be predominantly driven by ambient immune setpoints of each organ (Figs. 2B and 3B). We validated this cell-specific association via public genomic data by combining 83 pairs of colorectal cancer primary and metastatic tumors from five Gene Expression Omnibus datasets. Relative abundance of each immune cell population was estimated using the CIBERSORT cell deconvolution algorithm (49). In this analysis, activated CD4 memory T cells showed the second highest concordance (behind eosinophils) between primary tumor and liver metastases (Supplementary Fig. S11B). These results suggest that CD4 T cells, which may represent tumor-specific T cells, may traffic between the primary tumor and liver metastases.

### Liver and Peritoneal Metastases Exhibit Increased Immune Suppression Compared with Lung Metastases

**FIGURE 7:**
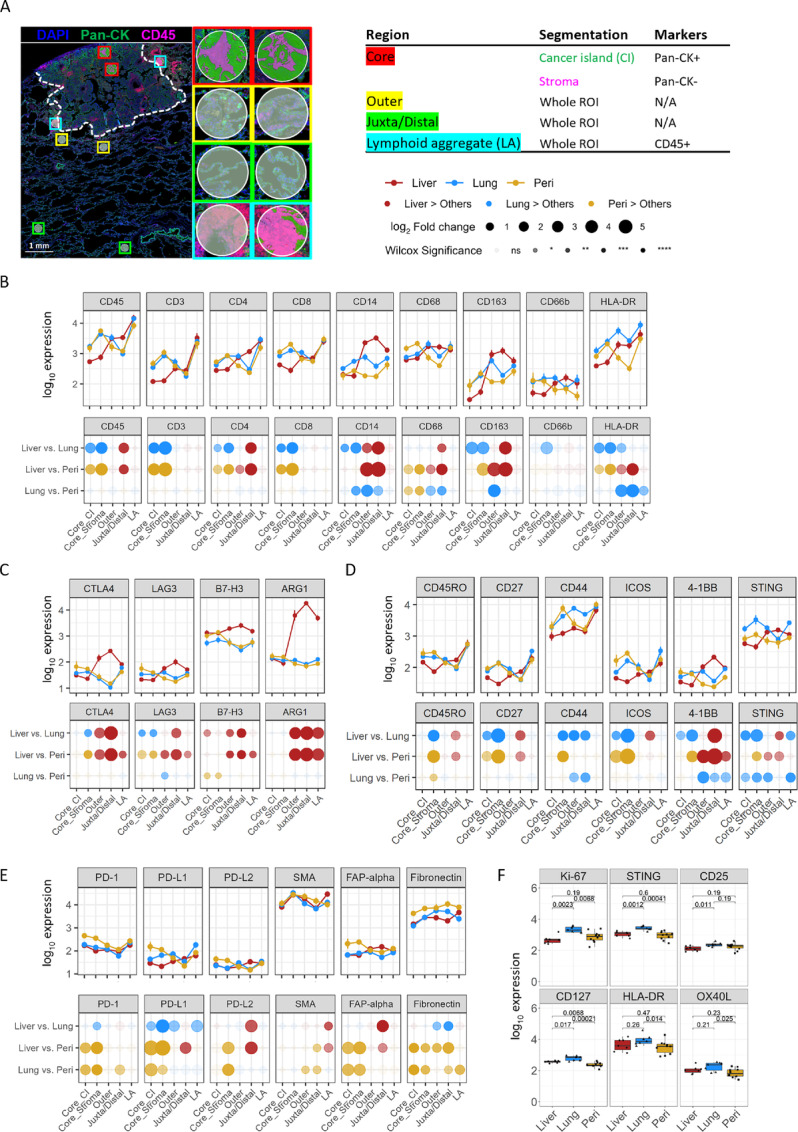
Higher immune activity in lung metastasis, while more immune suppressive in the liver and peritoneal metastases. **A,** Representative image of ROI selection in GeoMx digital spatial profiling from a colorectal cancer lung metastasis. Cancer island and stroma within tumor core were identified by the expression of Pan-CK. Cell identity markers from metastatic colorectal cancer tumors (**B**) and markers highly expressing in the liver (**C**), lung (**D**), and peritoneal (**E**) metastases in GeoMx assay. Paired comparison of gene expression between each two organs in the different histologic regions shown in bottom row. **F,** GeoMx markers expressing in the LA. Statistical significance was determined by Wilcoxon signed-rank test for B–F.

To further investigate potential mechanisms regulating organ-specific immune cell distribution in the metastatic TME, we utilized GeoMx digital spatial profiling to explore features of immune activation versus suppression within histologically distinct regions (Fig. 7A; Supplementary Fig. S12). We first compared whether the distribution of cell identity markers were consistent with our cell density analysis from mIF staining (Fig. 7B). Compared with lung and peritoneal metastatic tumors, expression of leukocyte (CD45) and T-cell (CD3, CD4, and CD8) markers in liver metastasis were lower in the tumor core but comparable in the peritumoral area. In addition, monocyte/macrophage markers (CD14, CD68, and CD163) were also lower in the tumor core of liver metastases, but CD14 and CD163 were more abundant in peritumoral regions of liver metastases than lung or peritoneal metastases. Finally, expression of CD66b (neutrophil marker) suggested comparable neutrophil infiltration across all organ sites, while HLA-DR expression (APC marker) indicated more abundant APC in lung metastases (Fig. 3B and C). Overall, the expression patterns of cell identity markers were largely consistent with cell distribution characterized by mIF assay, thus cross-validating the accuracy of both quantification methods.

We further interrogated metastatic site–specific features of the TME via expression of various biomarkers. In liver metastases, the immunosuppressive checkpoints CTLA-4, LAG-3, B7-H3, and ARG1were highly expressed within the outer invasive margin and distal region, but their levels were lower or similar inside the tumors compared with other metastatic tumor sites (Fig. 7C). In contrast, memory T-cell markers and costimulation makers, including CD27, CD44, CD45RO, ICOS, and 4-1BB, were lower within the tumor core of liver metastases, indicating decreased immune activity, possibly contributing to decreased lymphocyte infiltration (Fig. 7D). We found higher expression of STING in lung metastases, especially within LAs (Fig. 7D and F), suggesting increased cGAS-STING signaling in the lung metastatic TME. Expression of PD-1 and fibroblasts-related genes (SMA, FAP-alpha, and Fibronectin) were higher in peritoneal metastases than in liver and lung metastases (Fig. 7E). These findings are consistent with recent reports that fibrosis can prevent lymphocyte infiltration in colorectal cancer peritoneal metastases (20). Finally, we observed higher expression of markers of cell proliferation (Ki-69), T-cell activation, and antigen presentation in LAs surrounding lung metastases (Fig. 7F). Overall, these results demonstrate a connection between peritumoral immune activation and tumor penetration of lymphocytes into lung metastases, whereas liver and peritoneal metastases demonstrate an increased peritumoral immune population that associates with reduced tumor penetrance.

## Discussion

Patients with metastatic MSS colorectal cancer have demonstrated heterogeneous responses to immunotherapy. Recent studies from our group and others have indicated the metastatic site may predict clinical outcomes to immunotherapy—patients with liver metastases responding poorest. Thus, identifying organ-specific features that promote or suppress antitumor immunity may enable improved immunotherapy strategies for patients with MSS colorectal cancer. To our knowledge, this is the first study to directly compare the immune contexture across MSS colorectal cancer primary tumors, lung metastases, liver metastases, and peritoneal metastases. While other studies have identified novel transcriptional features of liver metastasis TME immune cell subsets (50, 51), comprehensive evaluation of immune cell localization and cell–cell interactions has not been done in metastatic MSS colorectal cancer. Our spatial approach of using a combination of mIF and digital spatial profiling provides a new understanding of the immune TME composition across primary tumors and major metastatic sites in MSS colorectal cancer.

Our findings reveal that abundance and spatial distribution of different immune subsets within colorectal cancer metastases vary in an organ-specific manner. Lung metastases contain similar densities and tumor infiltration by lymphocyte subsets as primary colorectal cancer tumors. In contrast, liver metastases show remarkably poor infiltration by lymphocytes into the tumor core, despite peritumoral densities similar to lung metastases and primary tumors. We also show that APC densities and APC–T cell interactions are higher in primary colorectal cancer tumors and lung metastases as compared with liver metastases. These findings demonstrate the importance of localized immune microenvironments in determining tumor infiltration by lymphocytes. In patients with cancer treated with immune checkpoint inhibitors, presence of mature TLSs, but not immature TLSs, was associated with improved objective response and improved progression-free survival and overall survival (41). Development of TLS reflects the functional capacity of antitumor immunity and intensity of cross-talk between tumor cells and lymphocytes. In this study, we observed a spectrum of immune structures, including small LAs composed only of T cells and B cells, to large fully mature TLS seen only in/around primary tumors. Mature TLSs were absent from all investigated metastatic sites, which could be due to impaired antigen presentation resulting from additional genetic alterations in metastatic tumor cells, altered/increased immunosuppressive signal in metastatic sites, or insufficient time for TLS to fully develop. Smaller LAs were more prevalent within lung metastases in comparison with liver and peritoneal metastases, identifying LAs as a potential key step in tumor localized immune activation.

Liver and lung metastases showed the greatest differences in immune activation versus suppression. The immunosuppressive environment of liver metastases is uniquely manifested within the peritumoral region. Checkpoint receptors on T and natural killer (NK) cells, including CTLA-4 and LAG-3, were highly expressed even in tumor-adjacent and normal tissues of liver, coincident with higher levels of B7-H3 and ARG1. The immune checkpoint protein B7-H3 expressed by nonmalignant cells can directly suppress adaptive immunity (52, 53); ARG1, which is highly expressed in hepatocytes, depletes l-arginine within the liver, thus depleting a key nutrient required for T-cell proliferation and memory formation (54, 55). Lung metastases, on the other hand, appeared to be more immunocompetent, expressing the highest levels of STING within the tumor core. The cGAS-STING cascade is primarily stimulated in tumor cells and/or DCs and is critical to sense tumor cell–derived cytosolic DNA signal, triggering type I IFNs secretion, induce TLS formation, and promote antitumor killing by T cells and NK cells (56, 57). Consistently, we found higher levels of T-cell activation markers CD27, CD44, ICOS, 4-1BB, and CD45RO in lung metastases compared with liver metastases. Peritumoral LAs in lung metastases also expressed high levels of STING signaling, cell proliferation, T-cell activation, and antigen presentation markers, consistent with previous reports that lung metastases have active exchange and cross-talk of T cells and APC with surrounding tissues (16). Peritoneal metastases represent yet another immune pattern. Although activation and suppression markers of T cells and NK cells were largely comparable between peritoneal and lung metastases, two main features distinguished peritoneal metastases from other metastatic sites: high levels of PD-1 receptor and ligands, and fibrosis-related proteins, both of which may be responsible for poor clinical outcome (19, 20).

Our study is limited by its relatively small size and needs further validation in a larger cohort, especially in the context of clinical outcomes. Nevertheless, our study characterizes the immune composition of TME at different metastatic sites and highlights potential pathways that could explain differences in immunotherapy response depending on the location of metastasis. These findings may elucidate why patients with advanced colorectal cancer without liver metastases respond better to immune checkpoint inhibitors in combination with or without VEGFR TKIs (4, 5, 7, 8, 10). Our results also suggest that remodeling of the immune TME may be required for response to immunotherapy for patients with liver or peritoneal metastases. Combining immunotherapy with chemotherapy or radiotherapy approaches has shown early initial promise in promoting local antitumor immunity (15, 58, 59). Furthermore, our findings suggest that targeted induction of the STING pathway may yield improved tumor local antigen presentation and T-cell activation. As such, specific targeting of CTLA-4, LAG-3, B7-H3, and arginase pathways within liver metastases, and inhibition of fibrosis in peritoneal metastases may be critical elements toward diminishing local immune suppression and enabling response to immunotherapy.

## Supplementary Material

Supplementary Table 1 Detailed summary of CRC specimens and patient overall survival.

Supplementary Figure 1 Characterization of TME in CRC primary and metastatic tumors with mIF.

Supplementary Figure 2 Characterization of immune cells in metastatic CRC TME by mIF.

Supplementary Figure 3 Distribution of immune cells in the cancer island and stroma of metastatic CRC TME.

Supplementary Figure 4 Distribution of PD-1+ T cells within the tumor core and peri-tumoral region in primarytumor.

Supplementary Figure 5 Distribution of lymphocytes in the cancer island, stroma and necrosis tissue within tumorcore and inner invasive region.

Supplementary Figure 6 Distribution of myeloid cells in the cancer island, stroma and necrosis tissue within tumor core and inner invasive region.

Supplementary Figure 7 Different antigen presentation potential CRC primary and metastatic tumors.

Supplementary Figure 8 Progressive maturation of TLS in CRC primary tumor.

Supplementary Figure 9 Progressive maturation of TLS in CRC primary tumor.

Supplementary Figure 10 Distribution of LA and TLS across colorectal cancer sites.

Supplementary Figure 11 Analysis of immune cells between paired primary and liver metastases.

Supplementary Figure 12 Clustered heatmap of relative expression of proteins (GeoMX DSP) per ROI from CRCmetastatic tumor specimens (5 tumors/metastatic site).

Graphic Summary Graphic Summary
