# Supplementary material for: Peritumoral Immune-suppressive Mechanisms Impede Intratumoral Lymphocyte Infiltration into Colorectal Cancer Liver versus Lung Metastases
Source: Cancer Res Commun. 2023 Oct 12;3(10):2082–95. doi: 10.1158/2767-9764.CRC-23-0212 (PMC10569153; doi:10.1158/2767-9764.CRC-23-0212)
Supplement: Supplementary Table 1 — Detailed summary of CRC specimens and patient overall survival. [file crc-23-0212-s01.pdf]

**Supplementary Table 1.** Detailed summary of CRC specimens and patient overall survival.

| Specimen type | Patient ID | Colon | Liver | Lung | Peri | OS Month | OS Status |
|---|---|---|---|---|---|---|---|
| Primary tumor only | 36 | Yes | | | | 69 | Alive |
| | 48 | Yes | | | | 20 | Deceased |
| Paired primary tumor and liver metastasis | 14 | Yes | Yes | | | 57 | Deceased |
| | 15 | Yes | Yes | | | 67 | Deceased |
| | 18 | Yes | Yes | | | 15 | Deceased |
| | 19 | Yes | Yes | | | 63 | Deceased |
| | 20 | Yes | Yes | | | NA | NA |
| | 21 | Yes | Yes | | | 39 | Deceased |
| | 22 | Yes | Yes | | | 44 | Deceased |
| | 23 | Yes | Yes | | | 42 | Deceased |
| | 24 | Yes | Yes | | | 40 | Deceased |
| | 25 | Yes | Yes | | | 34 | Deceased |
| | 39 | Yes | Yes | | | 60 | Alive |
| | 42 | Yes | Yes | | | 72 | Alive |
| | 44 | Yes | Yes | | | 80 | Deceased |
| Liver metastasis only | 35 | | Yes | | | 56 | Deceased |
| | 46 | | Yes | | | 82 | Deceased |
| | 47 | | Yes | | | 38 | Deceased |
| | 52 | | Yes | | | 37 | Deceased |
| | 53 | | Yes | | | 41 | Deceased |
| Paired primary tumor and lung metastasis | 1 | Yes | | Yes | | 28 | Alive |
| | 2 | Yes | | Yes | | 62 | Alive |
| | 3 | Yes | | Yes | | 51 | Alive |
| | 4 | Yes | | Yes | | 51 | Alive |
| | 5 | Yes | | Yes | | 16 | Deceased |
| | 6 | Yes | | Yes | | 77 | Alive |
| Lung metastasis only | 7 | | | Yes | | 66 | Deceased |
| | 8 | | | Yes | | 35 | Deceased |
| | 9 | | | Yes | | 50 | Deceased |
| | 10 | | | Yes | | 86 | Deceased |
| | 11 | | | Yes | | 61 | Deceased |
| | 40 | | | Yes | | 137 | Deceased |
| Peritoneal metastasis only | 27 | | | | Yes | 16 | Deceased |
| | 28 | | | | Yes | 20 | Deceased |
| | 29 | | | | Yes | 33 | Deceased |
| | 30 | | | | Yes | 40 | Deceased |
| | 31 | | | | Yes | 19 | Deceased |
| | 32 | | | | Yes | 25 | Deceased |
| | 33 | | | | Yes | 61 | Deceased |
| | 34 | | | | Yes | 25 | Deceased |
| Paired Liver and lung metastasis | 38 | | Yes | Yes | | 140 | Alive |
| | 51 | | Yes | Yes | | 27 | Deceased |
| **Subtotal #** | **42** | **21** | **20** | **14** | **8** | | |
