# Supplementary material for: Peritumoral Immune-suppressive Mechanisms Impede Intratumoral Lymphocyte Infiltration into Colorectal Cancer Liver versus Lung Metastases
Source: Cancer Res Commun. 2023 Oct 12;3(10):2082–95. doi: 10.1158/2767-9764.CRC-23-0212 (PMC10569153; doi:10.1158/2767-9764.CRC-23-0212)
Supplement: Supplementary Figure 1 — Characterization of TME in CRC primary and metastatic tumors with mIF. [file crc-23-0212-s02.pdf]

Supplementary Figure 1

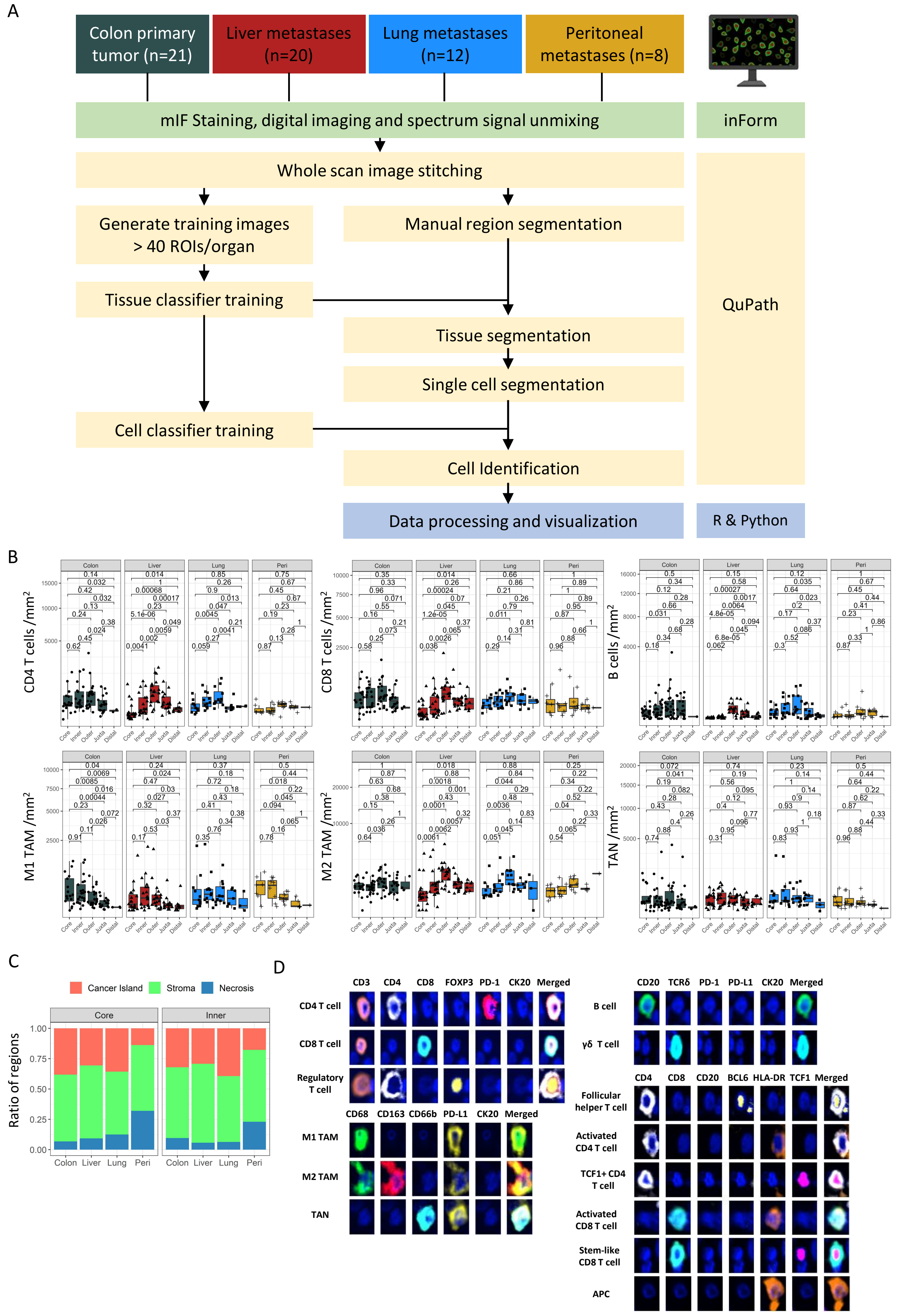

Supplementary Figure 1. Characterization of TME in CRC primary and metastatic tumors with mIF. (**A**). Workflow of metastatic CRC tumor sample staining and whole slide scanned image data process. (**B**). Bar plot of major immune cell types in different histopathologic regions of CRC primary tumors and metastases. Statistical significance was determined by Wilcoxon signed-rank test. (**C**). Proportion of tissue types (Cancer Island, Stroma, and Necrosis) in the core and inner invasive margin of CRC primary and metastatic tumors. (**D**). Representative images of cells identified by cell markers shown in Figure 1C.
