# Supplementary material for: Peritumoral Immune-suppressive Mechanisms Impede Intratumoral Lymphocyte Infiltration into Colorectal Cancer Liver versus Lung Metastases
Source: Cancer Res Commun. 2023 Oct 12;3(10):2082–95. doi: 10.1158/2767-9764.CRC-23-0212 (PMC10569153; doi:10.1158/2767-9764.CRC-23-0212)
Supplement: Supplementary Figure 2 — Characterization of immune cells in metastatic CRC TME by mIF. [file crc-23-0212-s03.pdf]

Supplementary Figure 2

A

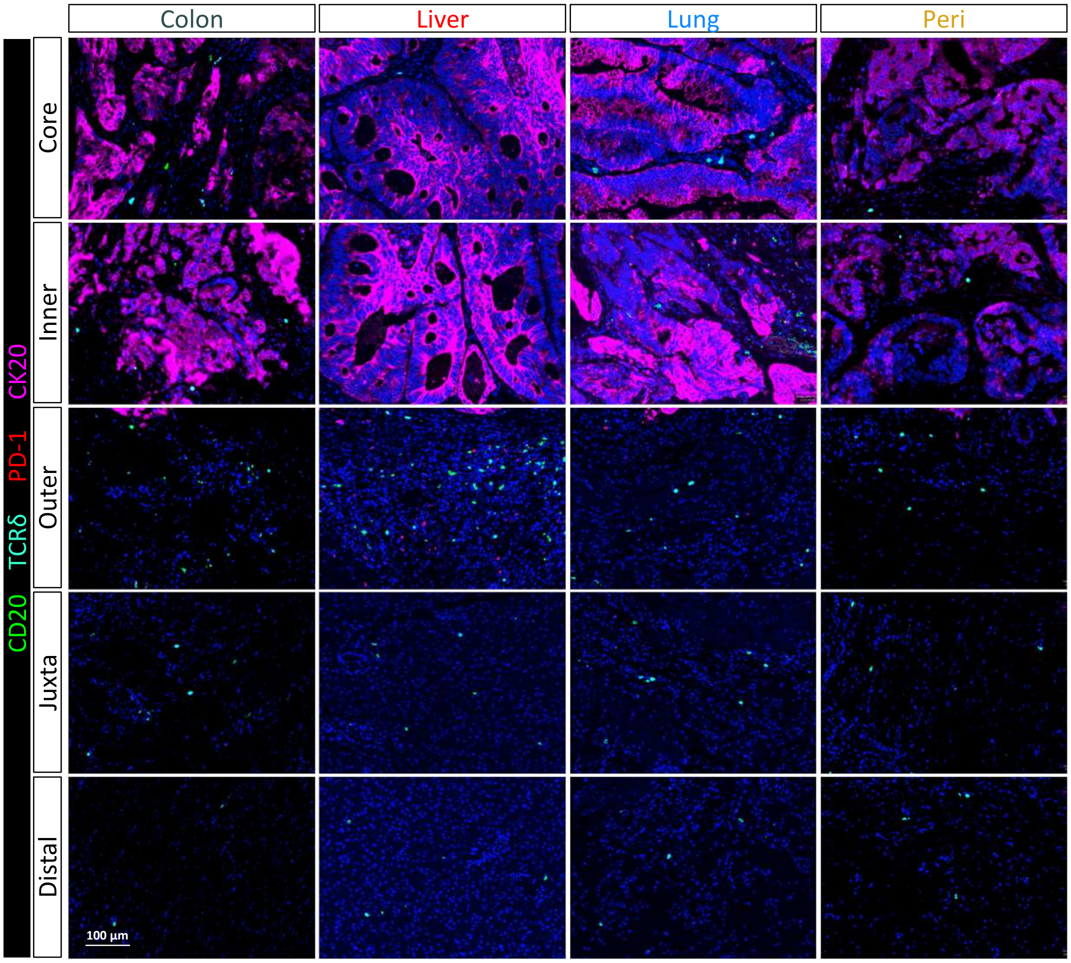

B

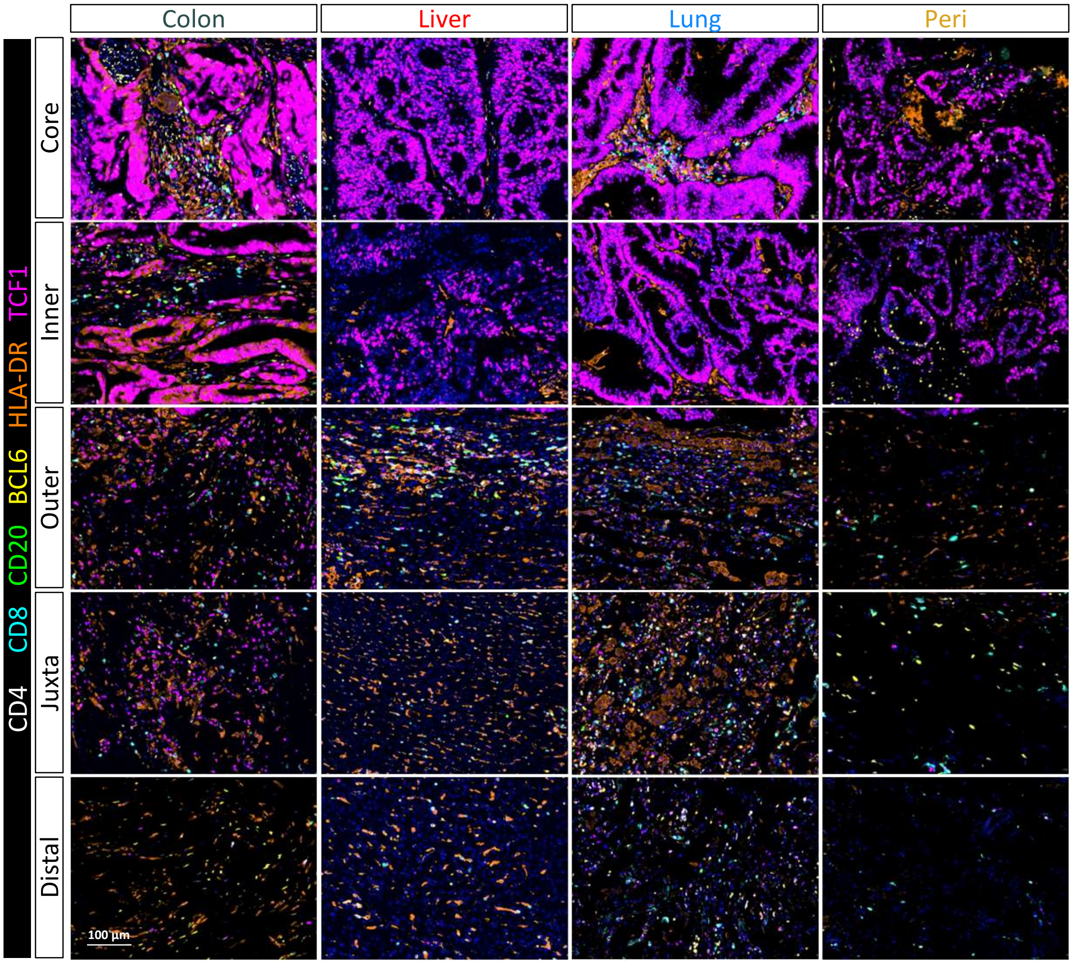

Supplementary Figure 2. Characterization of immune cells in metastatic CRC TME by mIF. (**A-B**). Representative images of primary and metastatic CRC tumors stained with mIF panel 3 (A) and panel 4 (B), five images in each column were selected from the same tumor.
