# Supplementary material for: Peritumoral Immune-suppressive Mechanisms Impede Intratumoral Lymphocyte Infiltration into Colorectal Cancer Liver versus Lung Metastases
Source: Cancer Res Commun. 2023 Oct 12;3(10):2082–95. doi: 10.1158/2767-9764.CRC-23-0212 (PMC10569153; doi:10.1158/2767-9764.CRC-23-0212)
Supplement: Supplementary Figure 3 — Distribution of immune cells in the cancer island and stroma of metastatic CRC TME. [file crc-23-0212-s04.pdf]

Supplementary Figure 3

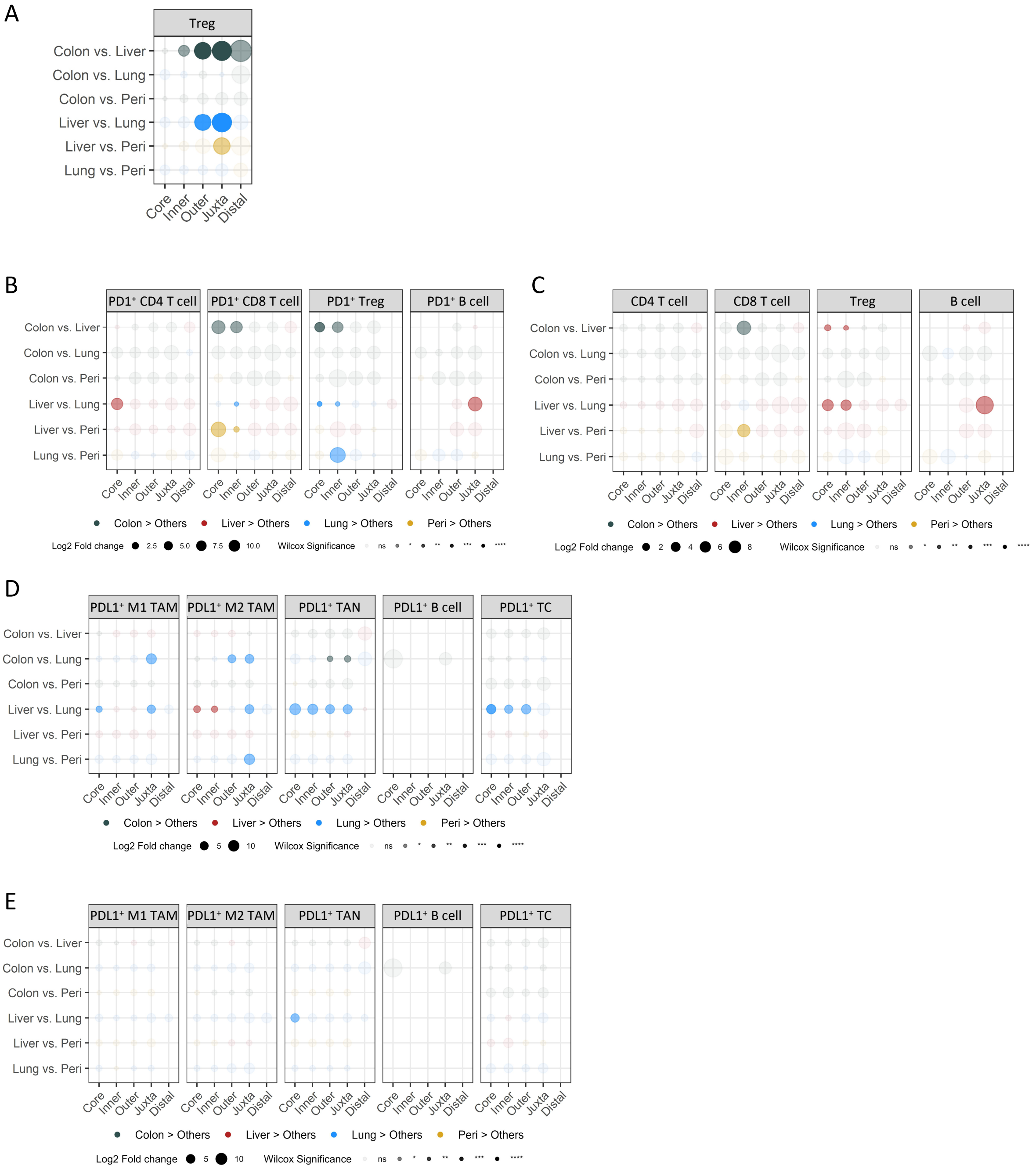

Supplementary Figure 3. Distribution of immune cells in the cancer island and stroma of metastatic CRC TME. (**A**). Paired comparison of percentage of Treg between each two organs in the different histological regions shown in Figure 2C. (**B-C**). Paired comparison of cell density (B) and percentage of PD-1 positive lymphocytes (C) between each two organs in the different histological regions shown in Figure 2D-E. (**D-E**). Paired comparison of cell density (D) and percentage of PD-L1 positive cells (E) between each two organs in the different histological regions shown in Figure 3C-D. Statistical significance was determined by Wilcoxon signed-rank test in (A-E).
