# Supplementary material for: Peritumoral Immune-suppressive Mechanisms Impede Intratumoral Lymphocyte Infiltration into Colorectal Cancer Liver versus Lung Metastases
Source: Cancer Res Commun. 2023 Oct 12;3(10):2082–95. doi: 10.1158/2767-9764.CRC-23-0212 (PMC10569153; doi:10.1158/2767-9764.CRC-23-0212)
Supplement: Supplementary Figure 4 — Distribution of PD-1+ T cells within the tumor core and peri-tumoral region in primarytumor. [file crc-23-0212-s05.pdf]

Supplementary Figure 4

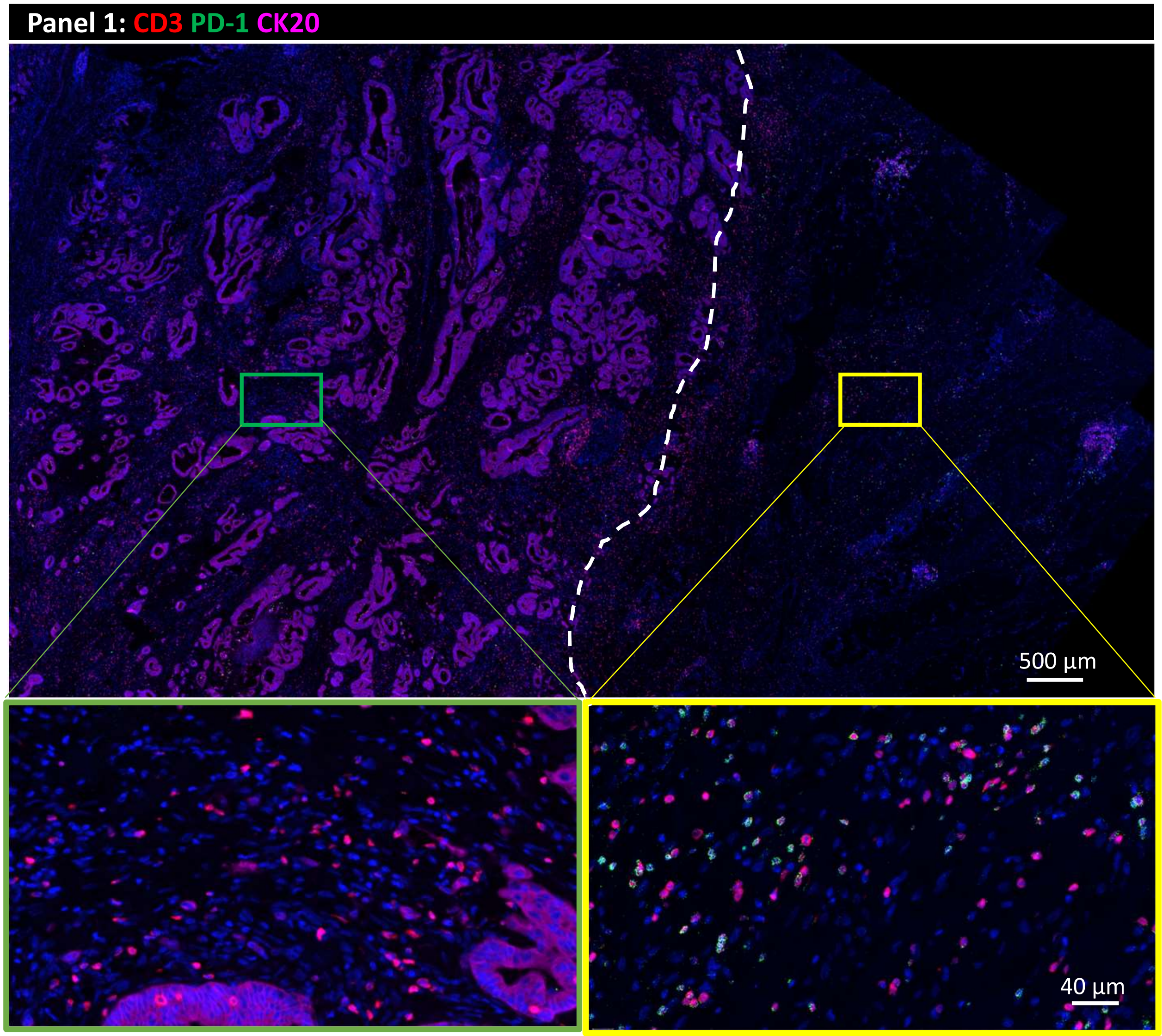

Supplementary Figure 4. Distribution of PD-1+ T cells within the tumor core and peri-tumoral region in primary tumor. Representative images of primary CRC tumor stained with CD3/PD-1/CK20.
