# Supplementary material for: Peritumoral Immune-suppressive Mechanisms Impede Intratumoral Lymphocyte Infiltration into Colorectal Cancer Liver versus Lung Metastases
Source: Cancer Res Commun. 2023 Oct 12;3(10):2082–95. doi: 10.1158/2767-9764.CRC-23-0212 (PMC10569153; doi:10.1158/2767-9764.CRC-23-0212)
Supplement: Supplementary Figure 6 — Distribution of myeloid cells in the cancer island, stroma and necrosis tissue within tumor core and inner invasive region. [file crc-23-0212-s07.pdf]

Supplementary Figure 6

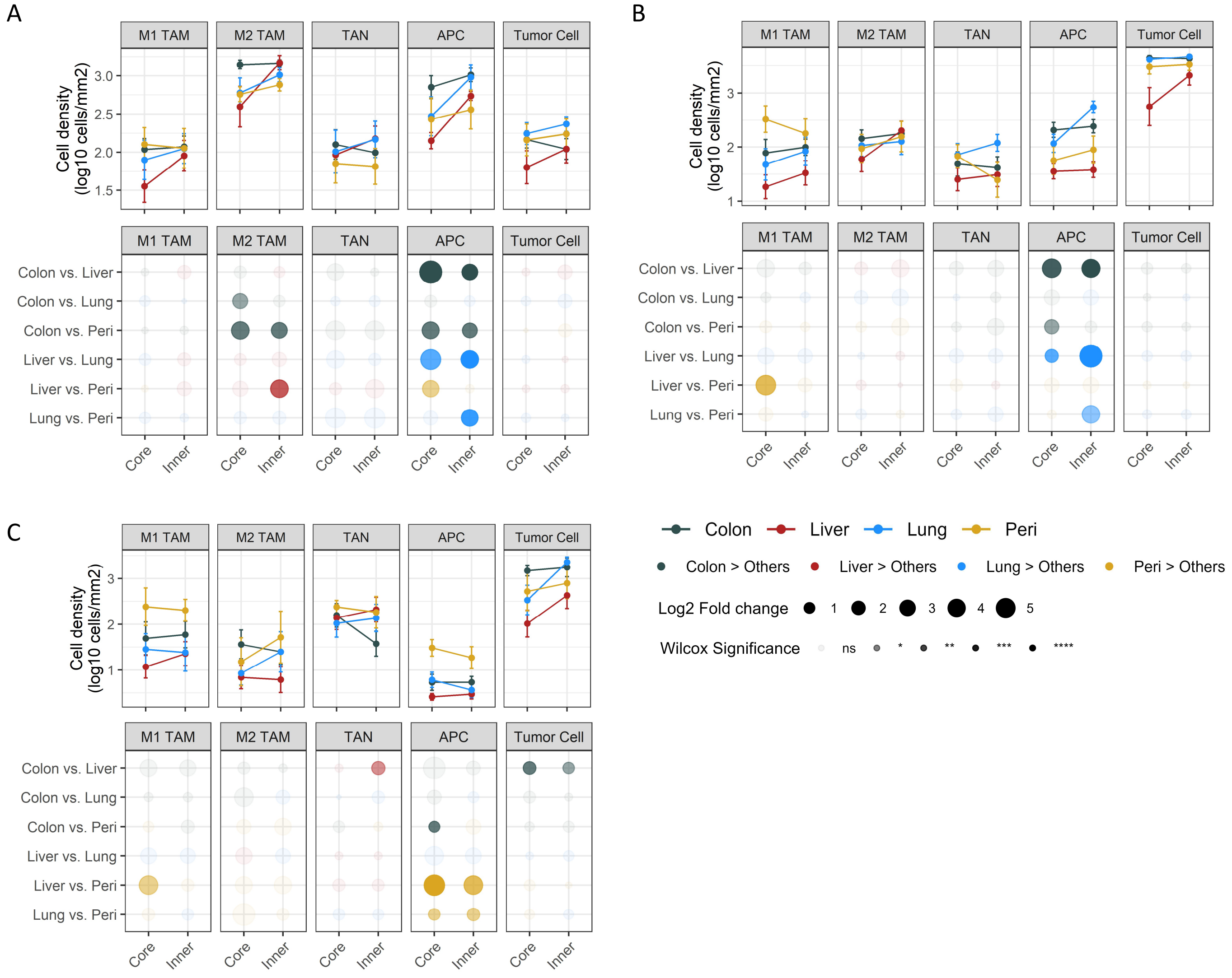

Supplementary Figure 6. Distribution of myeloid cells in the cancer island, stroma and necrosis tissue within tumor core and inner invasive region. (**A-C**). Cell density (log10 cells/mm^2) and paired comparison of cell density of myeloid cells in the stroma (A), cancer island (B) and necrosis (C) in CRC primary and metastatic tumors. Statistical significance was determined by Wilcoxon signed-rank test.
