# Supplementary material for: Peritumoral Immune-suppressive Mechanisms Impede Intratumoral Lymphocyte Infiltration into Colorectal Cancer Liver versus Lung Metastases
Source: Cancer Res Commun. 2023 Oct 12;3(10):2082–95. doi: 10.1158/2767-9764.CRC-23-0212 (PMC10569153; doi:10.1158/2767-9764.CRC-23-0212)
Supplement: Supplementary Figure 7 — Different antigen presentation potential CRC primary and metastatic tumors. [file crc-23-0212-s08.pdf]

Supplementary Figure 7

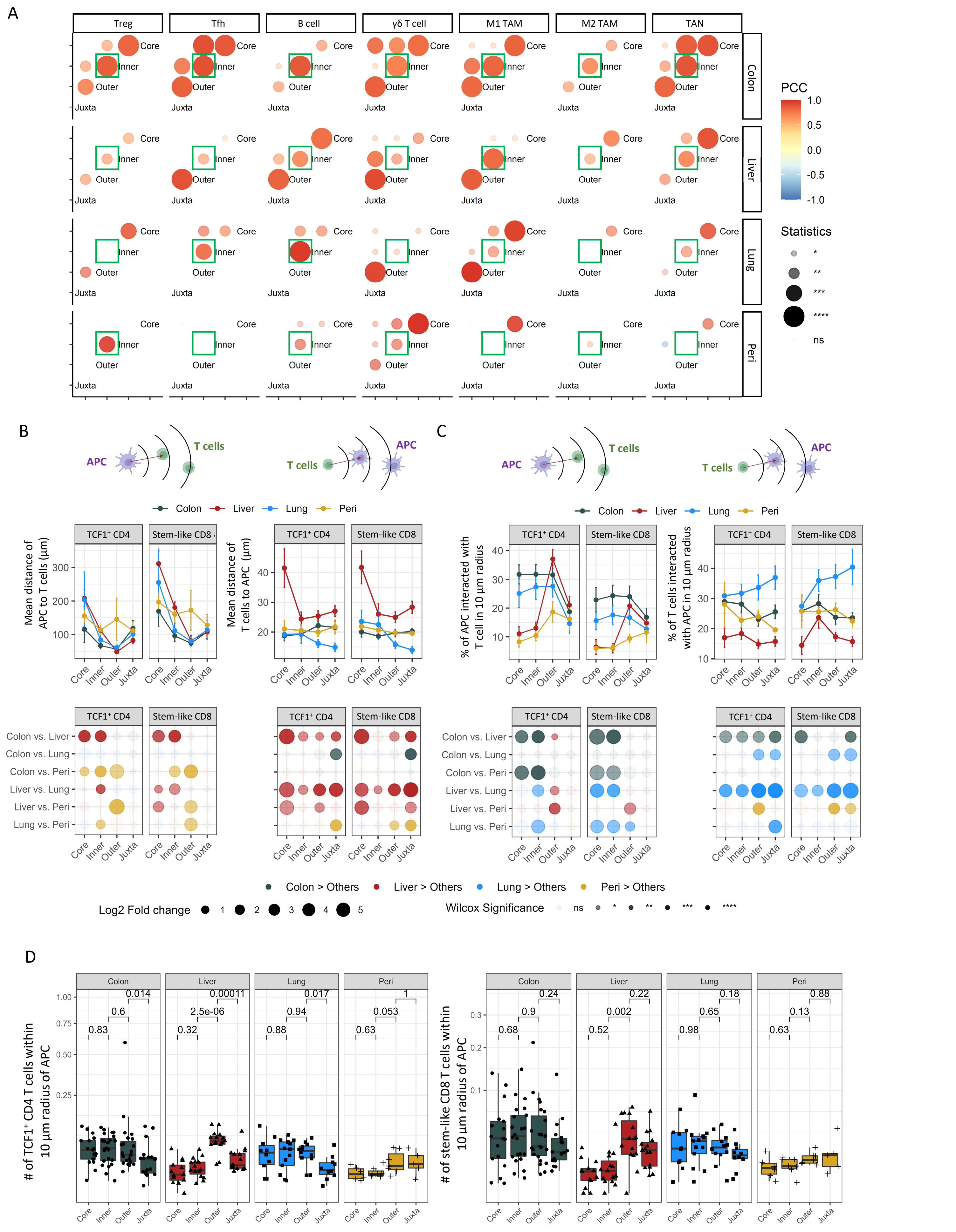

E

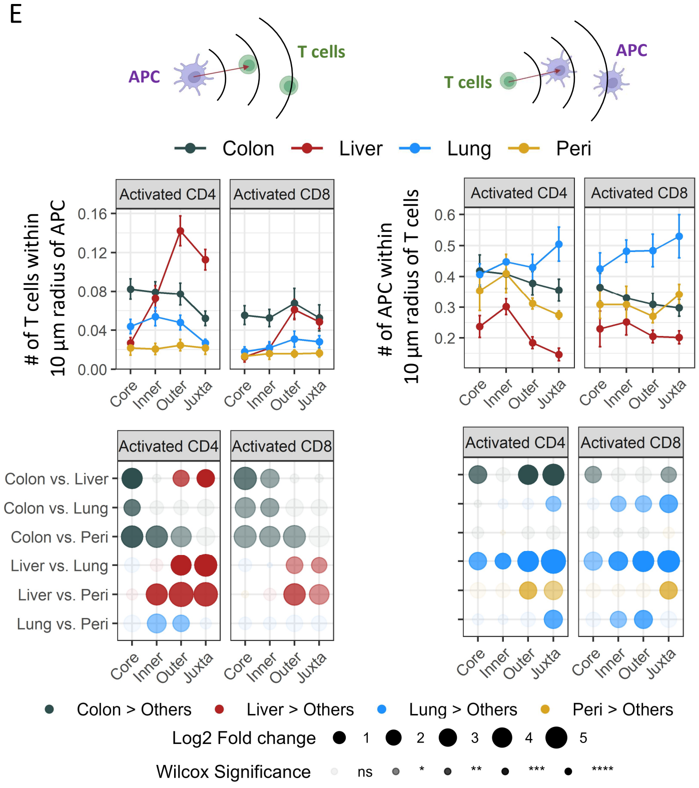

Supplementary Figure 7. Different antigen presentation potential CRC primary and metastatic tumors. (**A**). Correlations in the immune cell density between histological regions. Pearson correlation coefficient were calculated from the density of each immune cell type in different histological regions. (**B**). Mean distance of APCs to TCF1$^+$ CD4 T cells and stem-like CD8 T cells (left) and mean distance of TCF1$^+$ CD4 T cells and stem-like CD8 T cells to APCs (right). (**C**). Percentage of APCs directly interacted (within 10 µm radius) with TCF1$^+$ CD4 T cells and stem-like CD8 T cells (left) and percentage of TCF1$^+$ CD4 T cells and stem-like CD8 T cells directly interacted with APCs (right) in primary and metastatic tumors. (**D**). Mean number of TCF1$^+$ CD4 T cells and stem-like CD8 T cells around 10 µm radius of APCs (left) and APCs around 10 µm radius of TCF1$^+$ CD4 T cells and stem-like CD8 T cells (right) in different histopathologic regions of CRC primary tumors and metastases. (**E**). Mean number of Activated CD4 and CD8 T cells around 10 µm radius of APCs and mean number of APCs around 10 µm radius of Activated CD4 and CD8 T cells. Statistical significance was determined by Wilcoxon signed-rank test.
