# Supplementary material for: Peritumoral Immune-suppressive Mechanisms Impede Intratumoral Lymphocyte Infiltration into Colorectal Cancer Liver versus Lung Metastases
Source: Cancer Res Commun. 2023 Oct 12;3(10):2082–95. doi: 10.1158/2767-9764.CRC-23-0212 (PMC10569153; doi:10.1158/2767-9764.CRC-23-0212)
Supplement: Supplementary Figure 8 — Progressive maturation of TLS in CRC primary tumor. [file crc-23-0212-s09.pdf]

Supplementary Figure 8

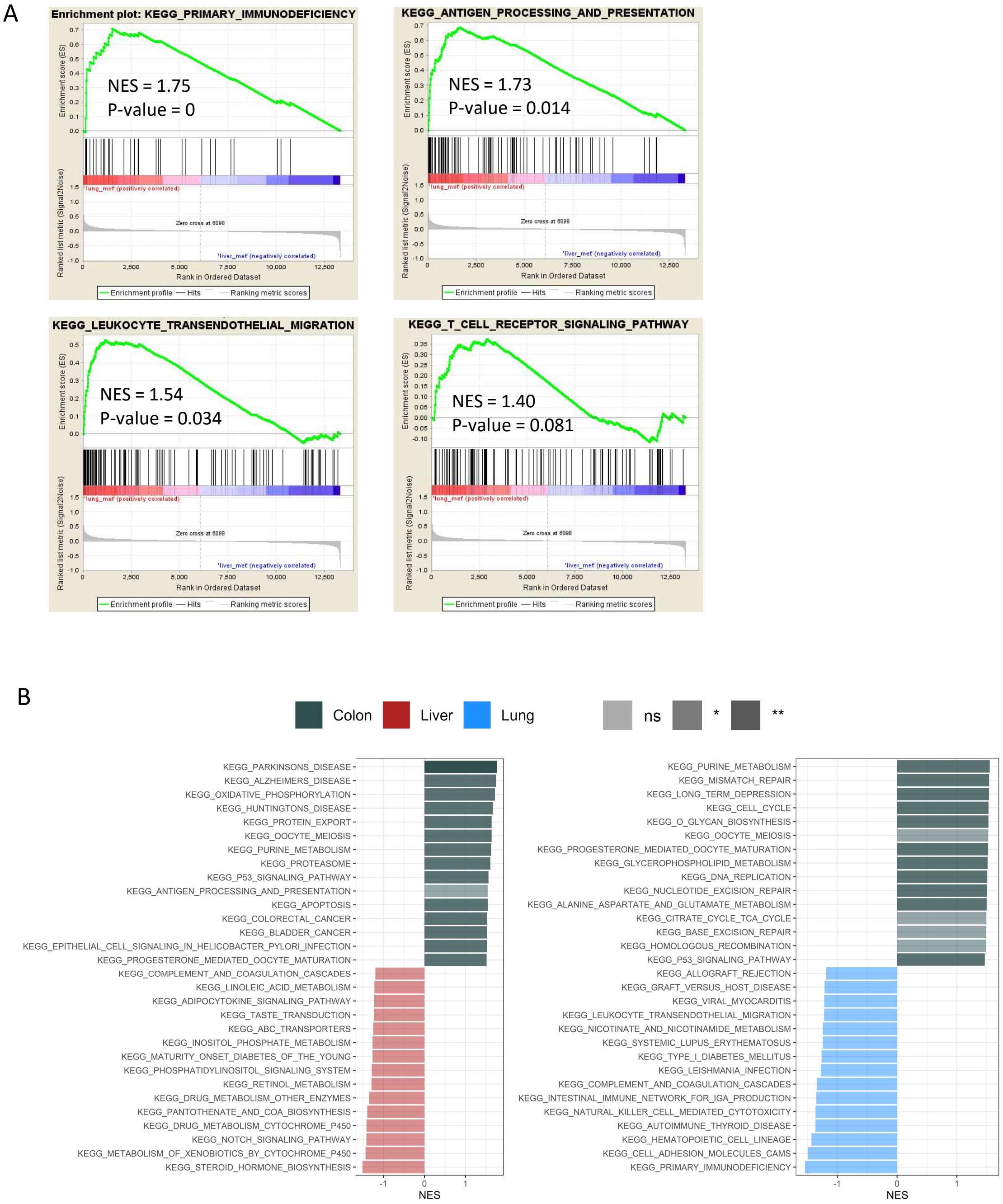

Supplementary Figure 8. Progressive maturation of TLS in CRC primary tumor. (**A**). Gene set enrichment analysis (GSEA) of liver and lung metastases samples from GES48468. (**B**). Gene set enrichment analysis (GSEA) of primary tumor and liver metastases samples (left) and primary tumor and lung metastases samples (right) from GES48468.
