# Supplementary material for: Peritumoral Immune-suppressive Mechanisms Impede Intratumoral Lymphocyte Infiltration into Colorectal Cancer Liver versus Lung Metastases
Source: Cancer Res Commun. 2023 Oct 12;3(10):2082–95. doi: 10.1158/2767-9764.CRC-23-0212 (PMC10569153; doi:10.1158/2767-9764.CRC-23-0212)
Supplement: Supplementary Figure 9 — Progressive maturation of TLS in CRC primary tumor. [file crc-23-0212-s10.pdf]

Supplementary Figure 9

A

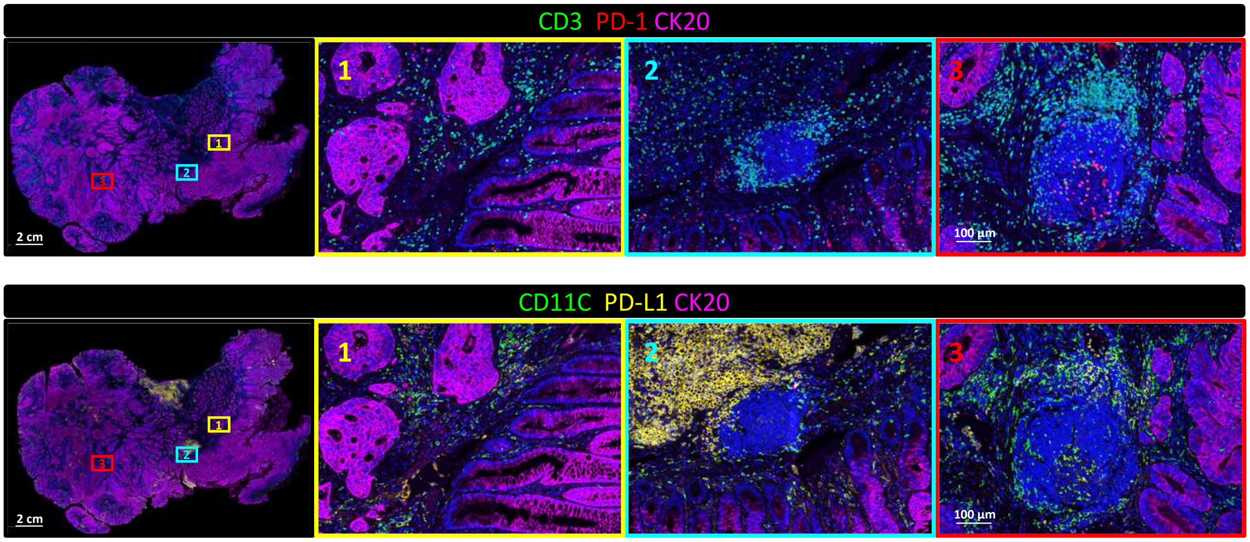

B

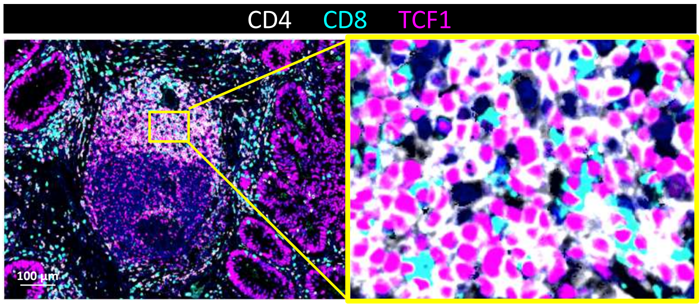

Supplementary Figure 9. Progressive maturation of TLS in CRC primary tumor. (**A**). Representative images of primary CRC tumor stained with CD3/PD-1/CK20 (top) or CD11C/PD-L1/CK20 (bottom). Numbered insets show higher magnification. (**B**). Magnified mature TLSs in primary CRC tumor as Figure 5A Box 3, highlighting CD4, CD8 and TCF1 markers.
