# Supplementary material for: Peritumoral Immune-suppressive Mechanisms Impede Intratumoral Lymphocyte Infiltration into Colorectal Cancer Liver versus Lung Metastases
Source: Cancer Res Commun. 2023 Oct 12;3(10):2082–95. doi: 10.1158/2767-9764.CRC-23-0212 (PMC10569153; doi:10.1158/2767-9764.CRC-23-0212)
Supplement: Supplementary Figure 10 — Distribution of LA and TLS across colorectal cancer sites. [file crc-23-0212-s11.pdf]

Supplementary Figure 10

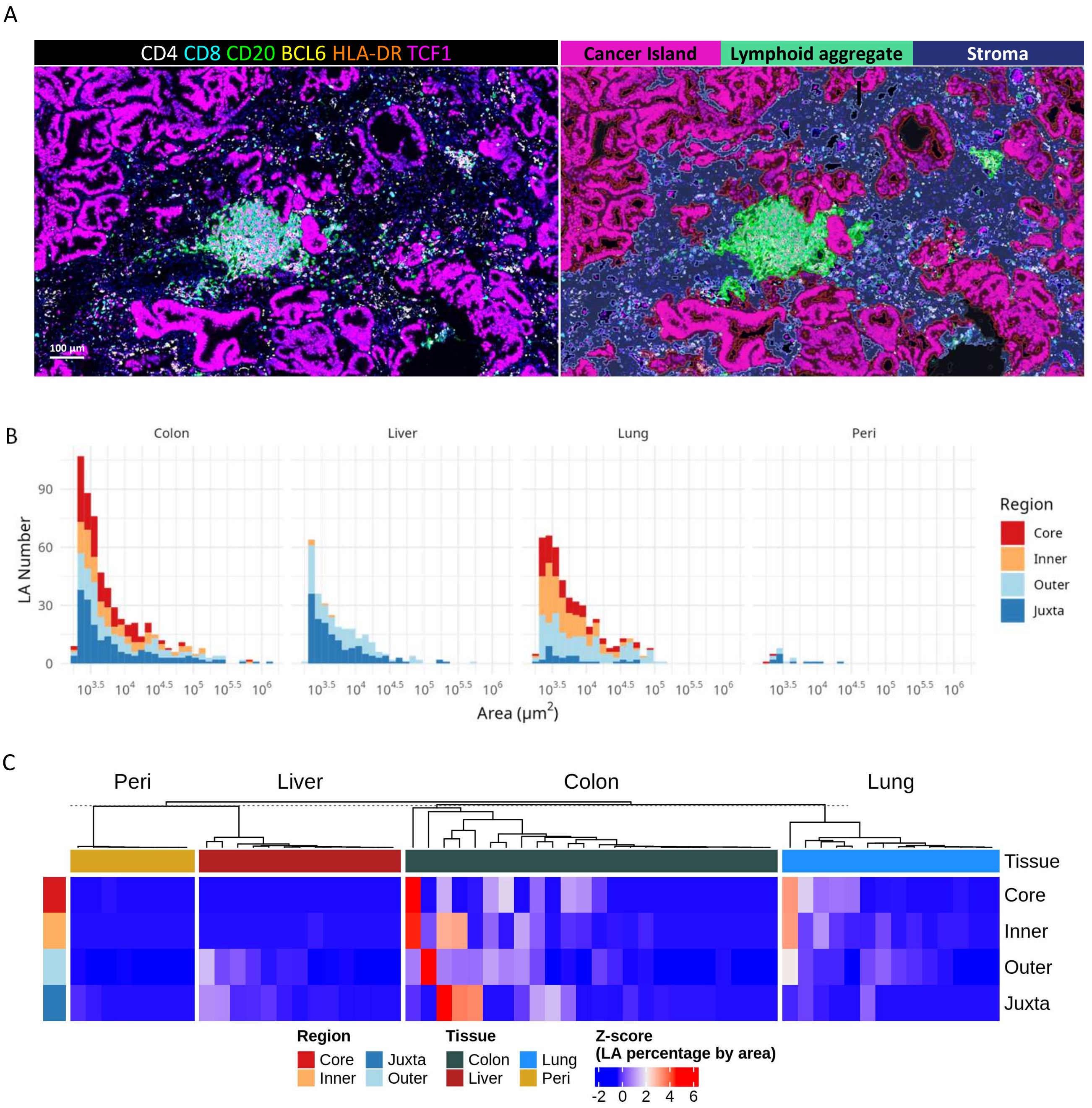

Supplementary Figure 10. Distribution of LA and TLS across colorectal cancer sites. (**A**). LA were identified with trained machine learning classifier. (**B**). Distribution of various size of LA in different histological regions of primary and metastatic tumors. (**C**). Heatmap of LA density from each analyzed specimen.
