# Supplementary material for: Peritumoral Immune-suppressive Mechanisms Impede Intratumoral Lymphocyte Infiltration into Colorectal Cancer Liver versus Lung Metastases
Source: Cancer Res Commun. 2023 Oct 12;3(10):2082–95. doi: 10.1158/2767-9764.CRC-23-0212 (PMC10569153; doi:10.1158/2767-9764.CRC-23-0212)
Supplement: Supplementary Figure 11 — Analysis of immune cells between paired primary and liver metastases. [file crc-23-0212-s12.pdf]

Supplementary Figure 11

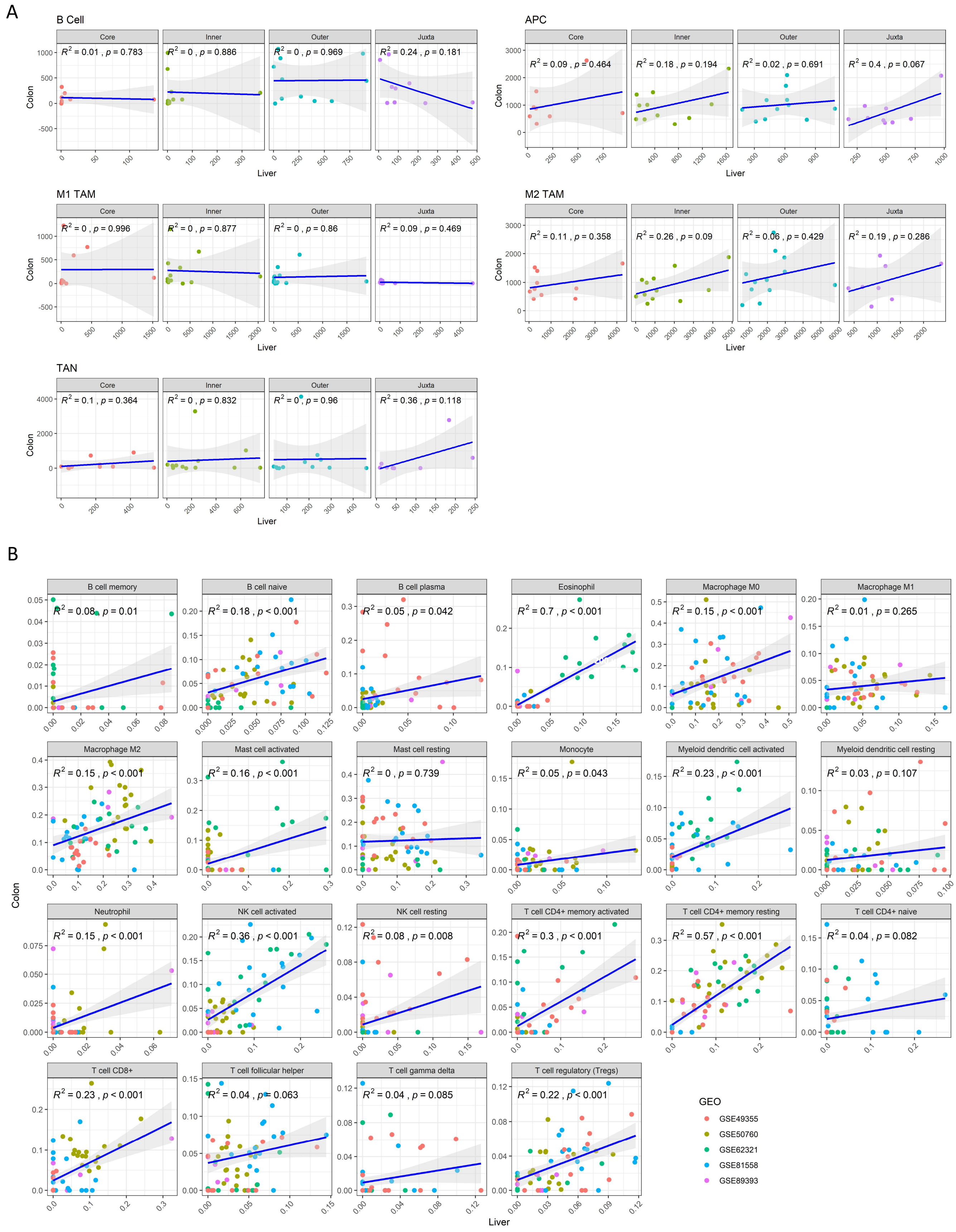

Supplementary Figure 11. Analysis of immune cells between paired primary and liver metastases. (**A**). Correlations of B cells, macrophage, neutrophils and APCs in paired primary and liver metastases. R^2 were calculated with the density of immune cells in different histological regions. (**B**). Correlations of immune cells between paired CRC primary tumor and liver metastases from 5 microarray datasets. The cell fractions from bulk tissue gene expression profiled were quantified by CIBERSORT.
