# Supplementary material for: Peritumoral Immune-suppressive Mechanisms Impede Intratumoral Lymphocyte Infiltration into Colorectal Cancer Liver versus Lung Metastases
Source: Cancer Res Commun. 2023 Oct 12;3(10):2082–95. doi: 10.1158/2767-9764.CRC-23-0212 (PMC10569153; doi:10.1158/2767-9764.CRC-23-0212)
Supplement: Supplementary Figure 12 — Clustered heatmap of relative expression of proteins (GeoMX DSP) per ROI from CRCmetastatic tumor specimens (5 tumors/metastatic site). [file crc-23-0212-s13.pdf]

Supplementary Figure 12

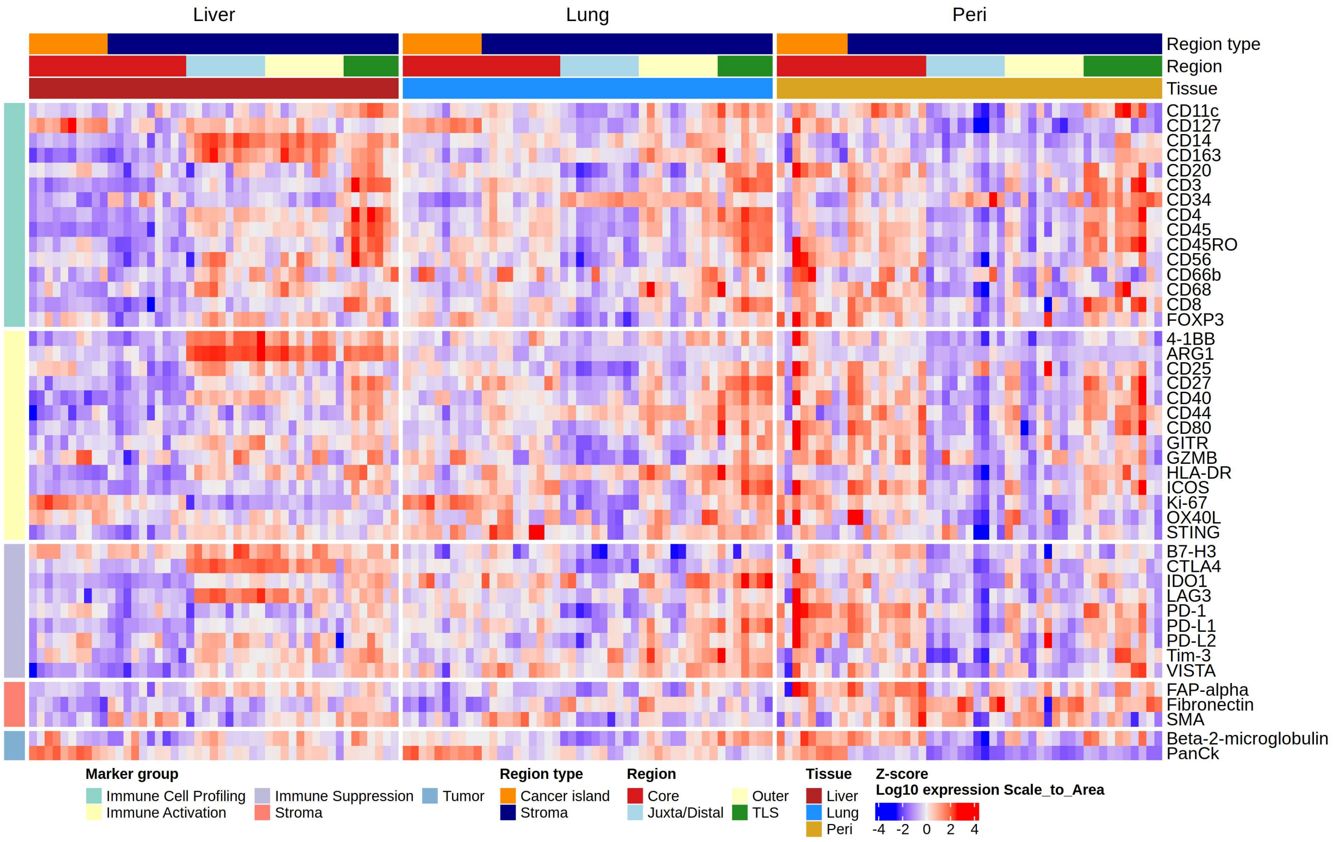

Supplementary Figure 12. Clustered heatmap of relative expression of proteins (GeoMX DSP) per ROI from CRC metastatic tumor specimens (5 tumors/metastatic site).
