# Supplementary figures and images for: Peritumoral Immune-suppressive Mechanisms Impede Intratumoral Lymphocyte Infiltration into Colorectal Cancer Liver versus Lung Metastases
Source: Cancer Res Commun. 2023 Oct 12;3(10):2082–95. doi: 10.1158/2767-9764.CRC-23-0212 (PMC10569153; doi:10.1158/2767-9764.CRC-23-0212)

# Graphic Summary

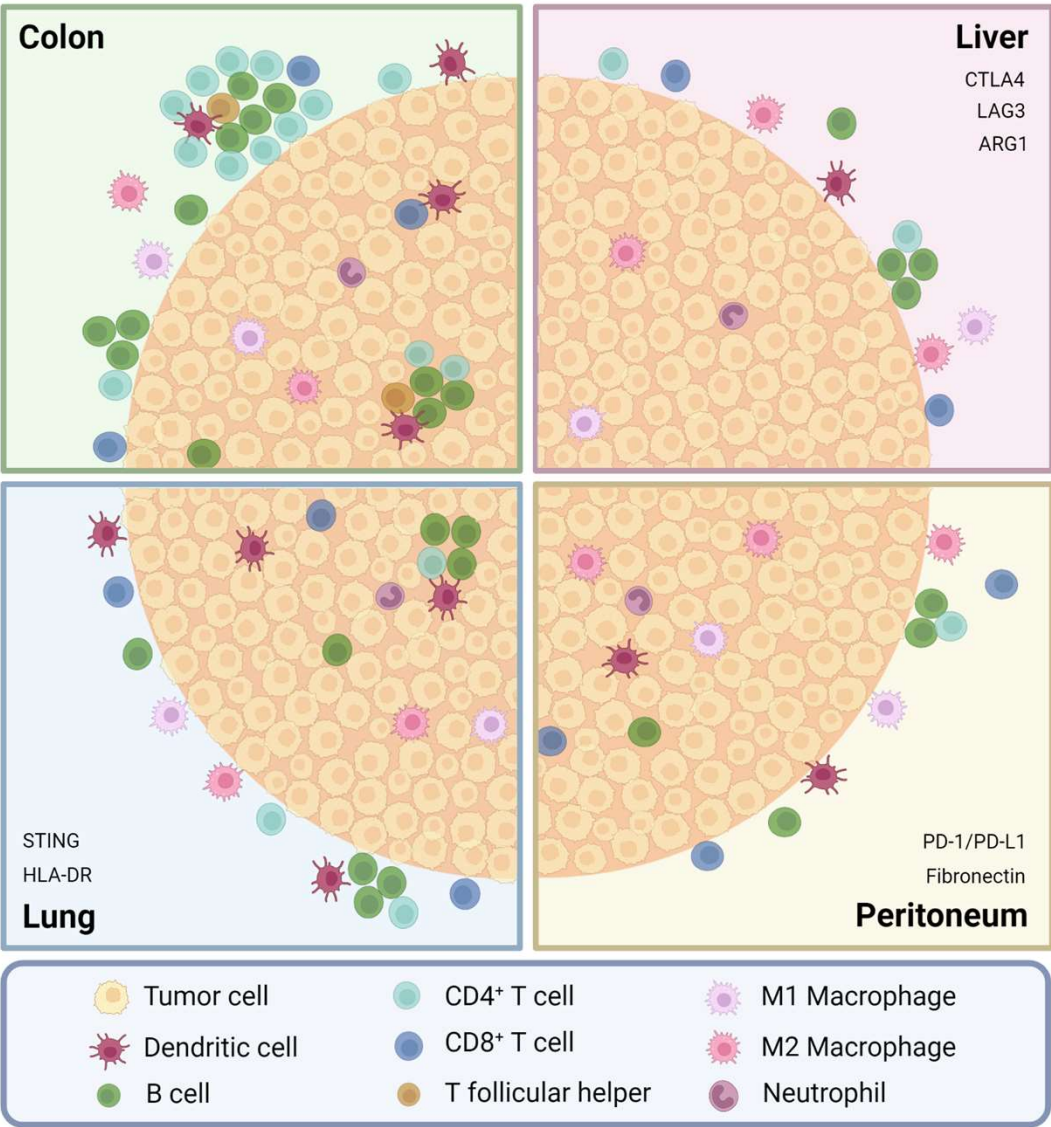

Supplement: Graphic Summary [file crc-23-0212-s14.pdf]
